# The Role of the Microtubule Cytoskeleton in Neurodevelopmental Disorders

**DOI:** 10.3389/fncel.2018.00165

**Published:** 2018-06-14

**Authors:** Micaela Lasser, Jessica Tiber, Laura Anne Lowery

**Affiliations:** Department of Biology, Boston College, Chestnut Hill, MA, United States

**Keywords:** cytoskeleton, microtubule dynamics, MAPs, neuronal migration, neurodevelopmental disorders

## Abstract

Neurons depend on the highly dynamic microtubule (MT) cytoskeleton for many different processes during early embryonic development including cell division and migration, intracellular trafficking and signal transduction, as well as proper axon guidance and synapse formation. The coordination and support from MTs is crucial for newly formed neurons to migrate appropriately in order to establish neural connections. Once connections are made, MTs provide structural integrity and support to maintain neural connectivity throughout development. Abnormalities in neural migration and connectivity due to genetic mutations of MT-associated proteins can lead to detrimental developmental defects. Growing evidence suggests that these mutations are associated with many different neurodevelopmental disorders, including intellectual disabilities (ID) and autism spectrum disorders (ASD). In this review article, we highlight the crucial role of the MT cytoskeleton in the context of neurodevelopment and summarize genetic mutations of various MT related proteins that may underlie or contribute to neurodevelopmental disorders.

## Introduction

The development of the central nervous system (CNS) and wiring of the brain is an extremely complex process, governed by the communication and careful coordination of the neuronal cytoskeleton, comprised of microtubule (MT), actin and intermediate filament networks (Menon and Gupton, 2016; Pacheco and Gallo, 2016; Kirkcaldie and Dwyer, 2017). Newly formed neurons face many challenges as they undergo dramatic changes in shape and migrate their way through the extracellular terrain in order to establish connections with other cells. Specifically, dynamic MTs play pivotal roles in creating cell polarity, as well as aiding in neural migration in order to establish appropriate neural connectivity throughout development and into adulthood. The elaborate MT network is integral to facilitate numerous morphological and functional processes during neurodevelopment, including cell proliferation, differentiation and migration, as well as accurate axon guidance and dendrite arborization. The organization and remodeling of the MT network is also essential for developing neurons to form axons, dendrites and assemble synapses. Moreover, in mature neurons, MTs continue to maintain the structure of axons and dendrites, and serve as tracks for intracellular trafficking, allowing motor proteins to deliver specific cargoes within the cell.

As brain development relies heavily on proper MT function, defects in the MT cytoskeleton can lead to detrimental effects on neural proliferation, migration and connectivity. Over the last several years, numerous studies have identified mutations within genes coding for proteins that interact with and directly modulate the structure and function of the MT cytoskeleton. Many of these MT-associated mutations have been linked to various neurodevelopmental disorders including lissencephaly, polymicrogyria, autism spectrum disorders (ASD) and intellectual disabilities (ID; Srivastava and Schwartz, 2014; Chakraborti et al., 2016; Stouffer et al., 2016). The regulation of the MT cytoskeleton during specific stages of brain development still remains an active topic of research. In this review article, we highlight various studies that illustrate important functions of the MT cytoskeleton that contribute to proper neural development and how genetic mutations within MT-related proteins can alter these crucial functions that may lead to disorders of neural development.

## The Role of the Microtubule Cytoskeleton During Neural Development

**Figure 1:**
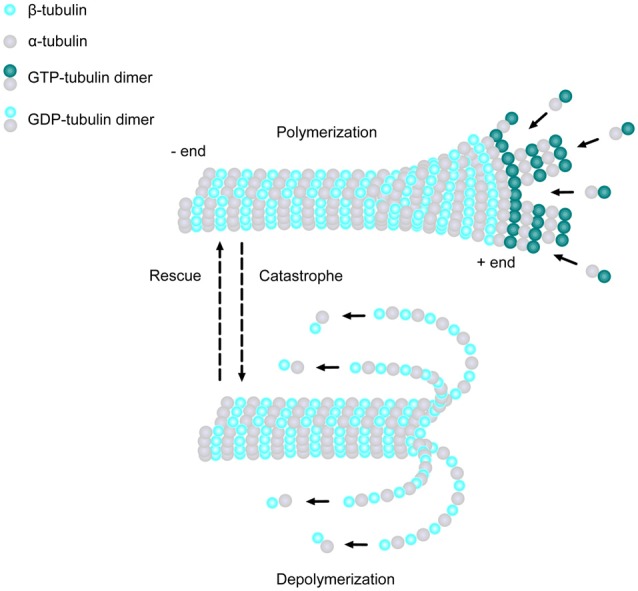
Microtubule (MT) basics. MTs are linear structures comprised of α-tubulin and β-tubulin heterodimers. MTs are extremely dynamic, existing in either a growing state (polymerization) or shrinking state (depolymerization), and can rapidly switch from growth to shrinkage (catastrophe) or from shrinkage to growth (rescue). Addition of new GTP-bound heterodimers occurs at the MT plus end during polymerization. Shortly thereafter, the tubulin subunits hydrolyze their bound GTP to GDP. When the addition of GTP-bound heterodimers slows and the MT lattice is composed of predominantly GDP-tubulin, the protofilaments splay apart and the MT depolymerizes.

MTs are one of the major cytoskeletal components present in all eukaryotic cell types. They are composed of α- and β-tubulin heterodimers, which bind to form 13 polarized linear protofilaments that associate laterally together to create the MT (Figure 1; Akhmanova and Steinmetz, 2008). MTs are extremely dynamic structures, existing in either a growing state (polymerization) or shrinking state (depolymerization). The plus ends of MTs can rapidly switch between these two states, going from growth to shrinkage (catastrophe), or from shrinkage to growth (rescue), a process called “dynamic instability” (Mitchison and Kirschner, 1984). Developing neurons depend on this stochastically dynamic nature of the MT cytoskeleton in order to remodel their shape, proliferate and migrate, as well as other processes during different phases of neural development, as described in more detail below.

### Neurite Formation and Axon Specification

Neurons begin their development as spherical, unpolarized cells, with MTs emanating from MT organizing centers (MTOCs), such as the centrosome, and are nucleated by the γ-tubulin ring complex (γ-TuRC; Kuijpers and Hoogenraad, 2011). This structure acts as a template for the α- and β-tubulin dimers to begin polymerization and is the cap of the minus end as the MT plus end grows away from the MTOC (Kuijpers and Hoogenraad, 2011). Once differentiation occurs, neurons form multiple processes, termed neurites, which extend from the spherical cell body and elongate to form thin protrusions (Götz and Huttner, 2005). MTs are one of the major players which influence the formation of neurites by creating small bundles that invade lamellipodia in multiple directions (Götz and Huttner, 2005). It has been suggested that MT sliding may play a key role in initiating neurite formation. One study demonstrated that MT-associated protein 2c (MAP2c) could stabilize MT bundles *in vitro*, which then rapidly move toward the cell periphery, prompting protrusions via a dynein-driven force (Dehmelt et al., 2006). It has also been shown that neurite formation can be induced by the MT motor protein, kinesin-1, which drives MT sliding and generates a mechanical force on the cell membrane (Lu et al., 2013; Winding et al., 2016). Studies have also demonstrated that actin filaments contribute to membrane protrusions, working in combination with stable MTs to initiate neurite outgrowth (Dent et al., 2011; Sainath and Gallo, 2015). Together, these results suggest that the local increase in actin dynamics, as well as the mechanical forces produced by MT sliding, all play key roles in stimulating neurite formation.

After neurite extension, one of the multiple processes becomes the axon, while the others later develop into dendrites (Menon and Gupton, 2016). Stable MTs are essential in axon specification and actively determine the polarity of developing neurons (Conde and Cáceres, 2009; Hoogenraad and Bradke, 2009). Interestingly, the ratio of stable to dynamic MTs was found to be significantly higher in one specific neurite compared to other neurites during this stage of neural development (Witte et al., 2008). Furthermore, axon formation was induced in unpolarized neurons following the addition of a photoactivatable analog of the MT-stabilizing drug taxol, while the addition of low doses of taxol led to the formation of multiple axons (Witte et al., 2008). These results suggest that in unpolarized neurons, the stabilization of MTs within one specific neurite occurs before axon formation begins. Once neurons begin to establish a distinct polarity, the centrosome progressively loses its function as an MTOC, with MTs beginning to nucleate from non-centrosomal MTOCs, such as Gogli outposts (Stiess et al., 2010; Stiess and Bradke, 2011; Ori-McKenney et al., 2012; Yau et al., 2014). Moreover, the stabilization of non-centrosomal MTs by the minus-end binding protein, calmodulin-regulated spectrin-associated protein 2 (CAMSAP2), was shown to be required for the establishment of neuronal polarity and axon formation (Yau et al., 2014). Reduction of CAMSAP2 inhibited both proper polarization and axon formation *in vitro*, and led to defects in neural migration *in vivo*, providing further evidence that MT stabilization is critical for these processes during neural development (Yau et al., 2014). Increased MT stabilization may also provide tracks for selective targeting of MT motor proteins to aid in the transportation of various organelles and proteins that are necessary for the eventual formation of axonal segments (Kapitein and Hoogenraad, 2011). Kinesin-1 has been shown to preferentially bind to stabilized MTs and accumulate in the future axon, contributing to early polarized trafficking, suggesting that an increase of stable MTs may lead to increased kinesin-mediated transport, driving both neuronal polarization and eventual axon specification (Nakata and Hirokawa, 2003; Jacobson et al., 2006).

### Axon Elongation and Branching

A major change that occurs, following axon specification, is the formation and enlargement of the growth cone, a dynamic structure at the tip of the growing axon responsible for driving axon elongation and branching (Kahn and Baas, 2016). Neuronal growth cones probe the extracellular environment and come into contact with various external stimuli, thus steering the axon in a particular direction (Lowery and Van Vactor, 2009). The growth cone uses the cytoskeletal machinery to progress through three distinct morphological stages, termed protrusion, engorgement and consolidation (Lowery and Van Vactor, 2009). To progress through these stages, an array of dynamic MTs penetrate into the central and peripheral domains of the growth cone, and can display different behaviors which include splaying, looping and bundling (Tanaka and Kirschner, 1991). This remodeling is essential for MTs to probe the growth cone periphery in search of guidance cues, which prompt growth cone advancement and turning. Additionally, growth cone formation and advancement require the interaction between actin filaments and dynamic MTs, which work in combination to promote the extension of lamellipodia and filopodia at the tip of the axon (Dent et al., 2011).

Cytoskeletal remodeling must also occur during axon branching, which involves an accumulation of actin filaments that will form axonal filopodia along the axon shaft (Mattila and Lappalainen, 2008). Shortly thereafter, MTs begin to invade the actin-rich filopodia with localized splaying of the normally bundled MT array (Dent et al., 1999; Gallo and Letourneau, 1999). This invasion by the axonal MTs into the filopodia allows their maturation into collateral branches as they continue extending. Early studies showed that there was an increase in the number of MTs at regions that eventually become axon branches, suggesting that MTs within the parent axon undergo fragmentation, and a portion of these fragments are then translocated to the developing branches (Yu et al., 1994; Kalil and Dent, 2014; Armijo-Weingart and Gallo, 2017). Thus, MT fragmentation and transportation appear to be key mechanisms which regulate the beginning of axon branch formation. Crosstalk between actin and MTs also seems to be required during the initial steps of axon branching (Dent and Kalil, 2001; Kalil and Dent, 2014; Gallo, 2016; Pacheco and Gallo, 2016). Dynamic MTs colocalize with F-actin in regions of axon branching, whereas stable MTs are excluded from these regions (Dent and Kalil, 2001). Moreover, when MT dynamics were dampened in neurons treated with either taxol or nocodazole, invasion of MTs into filopodia was reduced and they were unable to interact with actin-filament bundles, which resulted in decreased neurite formation (Dent et al., 2007). Recently, the cytoskeleton-associated protein, drebrin, was shown to regulate both actin filaments and MTs to initiate the formation of axon branches (Ketschek et al., 2016; Zhao et al., 2017). Endogenous drebrin was found to localize to axon actin patches *in vivo*, which eventually form axonal filopodia, suggesting that drebrin may contribute to the development of these precursor structures before axon branching (Ketschek et al., 2016). Additionally, reduction of drebrin severely inhibited axonal filopodia formation and the number of collateral branches *in vitro*, further demonstrating an essential role for drebrin in regulating these processes (Ketschek et al., 2016). Expression of drebrin also increased the number of axonal filopodia that contained end-binding protein 3 (EB3) comets, indicating that drebrin can promote the entry of dynamic MTs into axonal filopodia during the formation of axon branches (Ketschek et al., 2016). Thus, dynamic MTs and their interactions with actin filaments are crucial for the establishment and formation of collateral branches during neuronal development.

### Dendritogenesis and Synapse Formation

Following axon formation, other neurites begin to develop into dendrites, prompting dramatic changes to the MT network. While dendrites branch more extensively than axons, the behavior of MTs during this process has not yet been fully elucidated. One of the most striking features that distinguishes dendrites from axons is the orientation of their MTs. Axons display uniform plus-end distal MTs, while dendrites harbor a population of MTs with mixed polarity, where both plus and minus ends are oriented towards the cell body (Kapitein and Hoogenraad, 2015; Tas et al., 2017). These distinct polarity patterns are essential for determining the directionality of intracellular cargo transport, which maintains both the composition and morphological differences between axons and dendrites. Several studies have suggested that the translocation of MTs of differing orientations within dendrites plays a key role in establishing their mixed polarity (Sharp et al., 1995, 1997; Yu et al., 2000; Zheng et al., 2008; Rao et al., 2017). When MT assembly was inhibited with low levels of vinblastine, dendritic elongation was reduced, however MT reorientation was not hindered, suggesting that transport of MTs from the cell body is a possible mechanism for creating the non-uniform orientation of dendritic MTs (Sharp et al., 1995). Moreover, several studies have shown that neurons use distinct motor proteins to engage in specific transportation events that may drive this difference in MT orientation between axons and dendrites (Sharp et al., 1997; Yu et al., 2000; Zheng et al., 2008; Rao et al., 2017). Reduction of the kinesin-related motor protein CHO1 (also known as KIF23) from cultured neurons inhibited the movement of minus-end distal MTs into nascent dendrites and these processes failed to differentiate (Sharp et al., 1997; Yu et al., 2000). Likewise, reduction of kinesin-12 (also known as KIF15) produced similar phenotypic results (Lin et al., 2012). The abnormalities observed with depletion of kinesin-6 could be rescued by overexpression of kinesin-12, indicating that these two motor proteins may share functional redundancy within dendrites (Lin et al., 2012). Similarly, neurons treated with the dynein inhibitor, Ciliobrevin D, displayed a decrease in MT transport and abnormal MT orientation in the axon, which could be rescued after Ciliobrevin D washout (Rao et al., 2017). Thus, active MT transport by specific motor proteins is essential for both establishment and maintenance of MT bi-directionality during dendritic development.

The next steps in neuronal development are the formation and maturation of dendritic spines, which represent postsynaptic sites of excitatory synapses. The connections between an axon and a dendrite of neighboring neurons is a process that continuously occurs throughout development and into adulthood, as the brain rewires these connections in response to novel stimuli. For some time, it was thought that dendritic spines were devoid of dynamic MTs, and that actin was the main regulator of spine morphology and dynamics associated with synaptic plasticity. However, within the last decade, the use of new visualization techniques has revealed that MT dynamics do play an essential role in dendritic spine development (Yau et al., 2016; Dent, 2017). In concert with MT transport, MT polymerization actively occurs and contributes to the development of the dendritic branches. MT assembly occurs within the cell body, and after transport, these newly formed MTs are incorporated into the dendrite (Dent, 2017). Dynamic MTs can penetrate into dendritic spines of different shapes, including mushroom, stubby and thin, and can modulate their morphology by interacting with F-actin via +TIPs, such as EB3 (Gu et al., 2008; Jaworski et al., 2009; Dent, 2017). Furthermore, reduction of EB3 impairs spine development, and pharmacological manipulation of MT dynamics severely decreases the total number of spines and inhibits the formation of spines that are induced by brain-derived neurotrophic factor (BDNF; Gu et al., 2008; Jaworski et al., 2009). Interestingly, N-methyl-D-aspartate receptor (NMDAR)-dependent synaptic activation in hippocampal cell cultures increases the proportion of dendritic spines containing dynamic MTs, which contributes to spine enlargement, and this increase in MT invasion is inhibited by either blocking action potential activity or by dampening MT dynamics (Merriam et al., 2011). Additionally, dendritic spines exhibiting elevations in calcium signaling contain increased amounts of F-actin, and these spines are preferentially targeted by dynamic MTs, which interact with F-actin in a drebrin-dependent manner (Merriam et al., 2013). Moreover, studies have shown that the invasion of dynamic MTs into dendritic spines provide tracks for MT-dependent motors to deliver specific cargoes that are essential for synaptic plasticity (McVicker et al., 2016). It has also been demonstrated that the severing of dynamic MTs by the MT-severing proteins, katanin and fidgetin, are essential for dendrite development and synapse formation (Mao et al., 2014; Leo et al., 2015), which will be discussed in greater detail in subsequent sections. Together, these results strongly indicate that dynamic MTs are key regulators of dendritic spine formation, maintenance and synaptic activity, and that +TIPs play a role in this dynamicity to modulate spine morphology during neural development.

### Microtubules Guide Intracellular Transport During Axon and Dendrite Maintenance

MT-dependent cargo transport is an essential process that regulates the unique composition of both axons and dendrites and aids in maintaining their polarized morphology. This active transport mechanism is also necessary to accurately distribute specific cargoes and establish particular signaling pathways throughout the neuron. Neuronal intracellular transport is driven by the MT-dependent motors, kinesin and dynein, which move in opposite directions to deliver various cargo such as organelles, neurotransmitter receptors, cell signaling molecules and mRNAs, to the correct location within the cell (Hirokawa et al., 2010). Moreover, adaptor proteins and local signaling pathways regulate proper cargo delivery by aiding in cargo loading, anchoring and motility (Maday et al., 2014). The specific orientations of MTs are critical in determining the routes in which cargoes will travel throughout the neuron. For example, studies have demonstrated that dynein selectively transports cargoes along minus-end out oriented MTs found within dendrites (Kapitein et al., 2010; Tas et al., 2017). It has also been shown that kinesin-1 preferentially transports cargoes along MTs in axons, whereas kinesin-3 exhibits no selectivity, delivering cargoes to both axons and dendrites. It has been reported that specific post translational modifications of MTs are recognized by various kinesin superfamily members, which can regulate their localization and functions within neurons (Kapitein et al., 2010; Tas et al., 2017). As mentioned previously, it has been suggested that kinesin-1 preferentially binds to stabilized MTs that have either been acetylated or detyrosinated (Nakata et al., 2011), while kinesin-3 prefers to bind to tyrosinated MTs (Tas et al., 2017). However, the exact mechanisms which govern MT properties within axons and dendrites that subsequently guide specific motor proteins still remain unknown.

## Regulators of the Neuronal Microtubule Network

**Figure 2:**
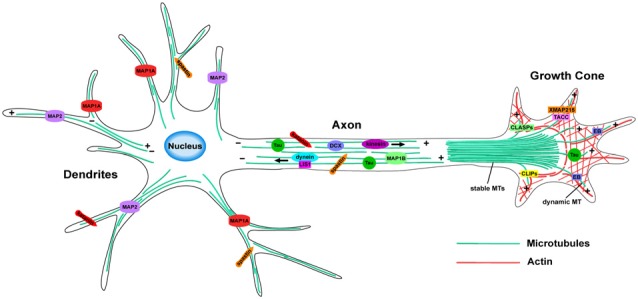
MT organization and MT-associated proteins (MAPs) in axons and dendrites. In axons, MTs form stable, polarized bundles, which provide structural integrity and serve as tracks to guide MT-dependent motor proteins. Axonal MTs are stabilized by several MAPs including Tau, MAP1B and DCX. The growth cone contains an array of both stable and dynamic MTs, which prompt growth cone advancement and turning. Various +TIPs accumulate at the growing MT plus ends in the growth cone, where they regulate MT dynamics during axon outgrowth and guidance. MTs of mixed polarity are located within dendrites where MAP1A and MAP2 aid in MT stabilization. The MT-severing proteins, katanin and spastin, are critical for reorganizing the MT network in both axons and dendrites.

The essential remodeling and organization of the MT cytoskeleton during neuronal morphogenesis relies on a vast array of MT-regulating proteins that have been identified over the last few decades (Figure 2). These proteins carry out specific functions to control MT dynamicity, fragmentation, stabilization, and intracellular transport. Many act directly on MTs to affect their nucleation, assembly, or stability, while others act indirectly by modulating tubulin levels or intracellular transport, producing downstream effects on neuronal differentiation. The combined efforts of MT-regulating proteins such as MT associated proteins (MAPs), +TIPs and MT motor proteins, provide the mechanisms by which the MT network reshapes its architecture during neuronal development. Here, we highlight several groups of MT regulators and their respective functions that control the assembly of new MTs and their dynamicity, as well as how they regulate MT stability, fragmentation and intracellular trafficking within neurons.

### Microtubule Plus End Tracking Proteins (+TIPs)

+TIPs are proteins which accumulate at growing MT plus-ends where they regulate MT dynamics, as well as facilitate their interactions with other proteins, organelles, and actin (Akhmanova and Steinmetz, 2008). Many families of +TIPs have been found to be crucial for different neurodevelopmental processes, specifically during neurite extension and axon outgrowth. For example, adenomatous polyposis coli (APC) is enriched in the nervous system where it plays a significant role in establishing neuronal polarity, as well as aiding in migration and axon navigation by stabilizing MTs (Shi et al., 2004; Koester et al., 2007; Eom et al., 2014). Other core regulators of the +TIP network involved in neural development are the EBs. EBs autonomously recognize the growing MT plus end, functioning as a scaffold for other +TIPs to associate and interact with the plus end, thereby regulating MT dynamics (Akhmanova and Steinmetz, 2008). Additionally, several studies suggest differential roles of EB proteins during neurodevelopment, demonstrating that the mechanisms underlying their regulation of the MT cytoskeleton are quite complex. For instance, as mentioned previously, EB3 expression is upregulated during neurodevelopment and is important for regulating MT dynamicity during dendritic spine development (Jaworski et al., 2008). Interestingly, this increase in EB3 expression coincides with a decrease in EB1 expression (Jaworski et al., 2008). However, EB1 is specifically upregulated during axon extension and has been shown to be critical for proper MT organization, as depletion of EB1 in *Drosophila* leads to abnormalities in MT architecture leading to aberrant axon outgrowth (Alves-Silva et al., 2012).

Cytoplasmic linker proteins (CLIPs) are another group of +TIPs that participate in several neural developmental processes including axon formation and outgrowth, as well as dendrite arborization (van de Willige et al., 2016). CLIPs are enriched in axonal growth cones, where they stabilize MTs invading the growth cone leading edge, and in dendritic spines, where they regulate the crosstalk between actin and dynamic MTs (Neukirchen and Bradke, 2011; Swiech et al., 2011). Additionally, mutations within CLIPs have been associated with ID (Larti et al., 2015), which will be discussed further in subsequent sections. Similar to CLIPs, cytoplasmic linker associated proteins (CLASPs) also regulate MT dynamics during axon outgrowth where they accumulate along MTs at the leading edge of the growth cone, suggesting a role in facilitating axon navigation during development (Lee et al., 2004; Marx et al., 2013). Additional +TIPs found to be important for regulating MT dynamics during neural development include the MT polymerase XMAP215 (Lowery et al., 2013), the XMAP215-interactor, TACC3 (Nwagbara et al., 2014; Bearce et al., 2015; Cammarata et al., 2016; Erdogan et al., 2017), as well as the other TACC family members, TACC1 and TACC2 (Lucaj et al., 2015; Rutherford et al., 2016).

### Microtubule Lattice-Binding Proteins

Remodeling of MTs within neurons is essential for the establishment and maintenance of neuronal polarity, axon outgrowth and guidance, synapse formation and migration. There are numerous MAPs that can bind along the MT lattice and regulate MT dynamics in order to properly organize and remodel the MT cytoskeleton during neural development. The most widely studied and best-characterized group of MT lattice-binding proteins are structural MAPs, which are comprised of three distinct groups including MAP1, MAP2 and tau.

There are three members of the MAP1 family found in vertebrates, MAP1A, MAP1B and MAP1S. MAP1B is highly expressed during early neuronal development and is found to be enriched at growing axons, where it regulates axon outgrowth via its interaction with the MT cytoskeleton (Bouquet et al., 2004; Jayachandran et al., 2016). Neuronal subtypes derived from *map1b*−/− mice revealed several defects including higher collateral axon branching, improper growth cone turning, as well as abnormalities in synaptic vesicle fusion and synaptic plasticity, suggesting that MAP1B is necessary during these processes (Bouquet et al., 2004; Bodaleo et al., 2016). Furthermore, reduction of MAP1B was also shown to decrease MT growth speed in the proximal and distal axon shaft, suggesting that MAP1B may function as a regulator of MT dynamics during axon outgrowth (Tymanskyj et al., 2012). Less is currently known about MAP1A, but it appears to participate in dendritic remodeling and preferentially localizes to postsynaptic densities (PSD) where it interacts with PSD proteins to support synaptic function (Koleske, 2013; Takei et al., 2015). It has also been shown that expression of MAP1A increases MT stability *in vitro* (Vaillant et al., 1998) and *Map1a* mutant mouse Purkinje neurons were shown to have abnormal MT networks, demonstrating an important role for MAP1A in MT organization and stabilization (Liu et al., 2015).

Similar to MAP1A, the MAP2 family of proteins are also enriched within dendrites and they have been shown to increase MT stability by rescuing catastrophes (Dehmelt and Halpain, 2005). Furthermore, they are important for regulating MT organization in dendrites by participating in MT bundling, as well as creating the proper spacing between MTs (Teng et al., 2001; Dehmelt and Halpain, 2005). Moreover, it was recently demonstrated that MAP2 can interact with both kinesin-1 and kinesin-3 motors to regulate their distribution of specific cargoes throughout the neuron (Gumy et al., 2017). MAP2-deficient mice show no overt neurological abnormalities, though they do display a reduction in MT density and dendrite length (Harada et al., 2002). However, mice deficient in both MAP2 and MAP1B have severe cortical defects, suggesting there may be a compensatory mechanism between these two proteins (Teng et al., 2001).

Tau is the most widely studied MT lattice binding protein due to its implication in neurodegenerative diseases (Matamoros and Baas, 2016). It is expressed in the brain throughout development into adulthood and appears to have several different functions in relation to MTs. Tau has been shown to increase MT polymerization, prevent MT depolymerization, and regulate MT organization (Wang and Mandelkow, 2016). Additionally, it has been demonstrated that tau dynamically interacts with MTs through a kiss-and-hop mechanism, where it remains associated with a single MT filament for only milliseconds before rapidly dissociating (Janning et al., 2014). Moreover, axonal transport rates are not significantly affected by either the elimination or overexpression of tau, further supporting the kiss-and-hop mechanism, as MT-dependent motor proteins would be impeded by tau if it associated with MTs for longer periods of time (Yuan et al., 2008; Janning et al., 2014). Tau is also expressed within axonal growth cones where it co-localizes with both MTs and actin filaments, and it can influence the organization of dynamic MTs to facilitate axon outgrowth and turning mediated by guidance cues (Li et al., 2014; Biswas and Kalil, 2018). Furthermore, tau seems to be critical for neuronal migration during brain development, as reduction of tau impaired the radial migration of cortical neurons, in addition to causing morphological abnormalities of the leading edge in these cells (Sapir et al., 2012). Interestingly, there may be functional redundancy between tau and MAP1B, as both have been shown to act synergistically to promote axon outgrowth and neuronal migration (Takei et al., 2000).

### Microtubule Severing Proteins

The reorganization of MTs depends on the process of MT severing, accomplished by enzymes such as katanin, spastin and fidgetin, which act to cut MTs into small pieces so that they can be transported to other locations within the cell in order to form a new MT array. Katanin consists of a P60 subunit, encoded by KATNA1, which functions to sever MTs, and a P80 subunit, encoded by KATNB1, which regulates the ATPase activity and localizes the protein primarily to centrosomes (McNally et al., 2000). In addition to the centrosome, katanin has been found to be highly expressed in growing axons (Karabay et al., 2004). Proper function of katanin within neurons seems to be dosage-dependent. Expression of a dominant-negative P60-katanin construct inhibited MT severing leading to abnormal axon outgrowth in cultured rat sympathetic neurons (Karabay et al., 2004). Conversely, overexpression of the P60-katanin construct led to increased MT severing, which was also detrimental to axon outgrowth (Karabay et al., 2004). More recently, katanin-mediated severing has been shown to regulate the development of dendrites and synapses at the neuromuscular junction (NMJ). The loss of the katanin P60 subunit in *Drosophila* resulted in abnormal synapse morphology at the NMJ, including an increased number of small boutons, more stable MT loops and dendritic overgrowth (Mao et al., 2014). Moreover, genetic mutations within several katanin-associated proteins have been linked to microcephaly, suggesting that proper function of katanin is important during early brain development (Bartholdi et al., 2014).

Similar to katanin, spastin is another MT severing protein that is highly enriched in developing axons and is thought to be involved in promoting axon outgrowth (Yu et al., 2008). Reduction of spastin function in zebrafish caused severe defects in motor axon outgrowth, as well as abnormalities in neuronal connectivity (Wood et al., 2006). Additionally, knockdown of spastin in cultured neurons reduced axonal branching, whereas overexpression increased the number of branches and filopodia, indicating that spastin also functions in a dosage-dependent manner (Yu et al., 2008). Furthermore, mutations within spastin cause autosomal dominant hereditary spastic paraplegia, which is a disease characterized by axonal degeneration that leads to a progressive gait disorder (Hazan et al., 1999). Therefore, the regulation of MT severing by spastin is vital for axon outgrowth that is necessary to establish proper neural connectivity.

Less is currently known about the precise functions of fidgetin, however it has been associated with various aspects of vertebrate embryonic development (Cox et al., 2000). It has been demonstrated that fidgetin can depolymerize and sever MTs *in vitro*, as well as regulate MT dynamics at mitotic centrosomes (Mukherjee et al., 2012). Moreover, knocking down fidgetin in *Drosophila* disrupts normal axon development by causing an increase in the number of synaptic boutons, as well as an increase in stable MTs (Leo et al., 2015). Interestingly, these results are similar to the phenotypes previously observed with the reduction of katanin, suggesting that fidgetin behaves in a similar fashion to other MT-severing enzymes (Mao et al., 2014). However, axons in cultured vertebrate neurons are significantly longer with more minor processes and contain an increase in labile MT mass when fidgetin is depleted, suggesting that fidgetin regulates axon elongation by severing MT labile domains during neurodevelopment (Leo et al., 2015).

## The Major Players of Neuronal Movement: Microtubule-Associated Genes Linked to Disorders of Neuronal Migration

Development of the brain requires extensive neuronal proliferation and migration. This orchestrated movement of neurons to their final destination within the brain relies heavily on the support and coordination of the MT cytoskeleton, along with various MT-associated proteins. Abnormalities in proliferation or migration can lead to downstream defects in neural connectivity and various neurodevelopmental disorders. Human and mouse genetic studies have been instrumental in understanding the dynamic nature and function of the MT cytoskeleton and its associated regulators during neuronal migration. In the following sections, we discuss several well-characterized neuronal migration disorders and the various MT-associated genetic mutations that lead to these disorders during brain development (Table 1).

**Table 1:** Microtubule (MT)-associated genes linked to neurodevelopmental diseases.

Disease	Gene	Major pathways/roles	Additional phenotypes	References
Intellectual disabilities (ID)	KIF1A	Kinesin; MT-dependent motor		Willemsen et al. (2014)
	KIF4A	Kinesin; MT-dependent motor		Kondo et al. (2012), Lee et al. (2015), Ohba et al. (2015) and McVicker et al. (2016)
	CLIP1	MT binding; +TIP; MT dynamics		Coquelle et al. (2002), Jaworski et al. (2008), Swiech et al. (2011) and Larti et al. (2015)
	KATNAL1	MT severing	Microcephaly	Bartholdi et al. (2014) and Banks et al. (2018)
	MID2	Ubiquitin ligase; MT binding		Geetha et al. (2014) and Gholkar et al. (2016)
Autism spectrum disorders (ASD)	AUTS2	Cytoskeletal remodeling		Kawauchi et al. (2003), Kalscheuer et al. (2007), Oksenberg et al. (2013) and Hori et al. (2014)
	ADNP	Chromatin remodeling	ID, developmental delay, motor delay	Mandel et al. (2007), Vulih-Shultzman et al. (2007), Oz et al. (2012), Helsmoortel et al. (2014), Schirer et al. (2014), Gozes et al. (2017a), Gozes et al. (2017b), Ivashko-Pachima et al. (2017), Gozes et al. (2018) and Van Dijck et al. (2018)
	JAKMIP1	MT-associated kinase; MT dynamics; GABA receptor trafficking		Couve et al. (2004), Steindler et al. (2004), Vidal et al. (2007), Kaminsky et al. (2011), Hedges et al. (2012), Poultney et al. (2013) and Berg et al. (2015)
	MARK1	MT-associated kinase; MT dynamics; mitochondrial trafficking		Drewes et al. (1997), Mandelkow et al. (2004), Maussion et al. (2008) and Sapir et al. (2008)
Microcephaly	ASPM	Mitotic spindle protein; cell division	ID, speech delay, seizures, short stature	Kouprina et al. (2005), Jiang et al. (2017) and Duerinckx and Abramowicz (2018)
	MCPH1	Mitotic spindle protein; DNA damage response; chromosome condensation	ID, seizures, short stature	Trimborn et al. (2004) and Gruber et al. (2011)
	STIL	Mitotic spindle checkpoint protein; centriole amplification	ID, seizures, short stature	Kumar et al. (2009), Kitagawa et al. (2011) and Vulprecht et al. (2012)
	CDK5RAP2	Centrosome integrity; spindle pole morphology	ID	Bond et al. (2005), Choi et al. (2010) and Lizarraga et al. (2010)
	CENPJ	Centrosome integrity; spindle pole morphology	ID, seizures	Kitagawa et al. (2011) and Garcez et al. (2015)
	PRUNE1	Cell motility; MT dynamics		Zollo et al. (2017)
	KIF20B	Kinesin; MT-dependent motor; cell polarity; cytokinesis		McNeely et al. (2017)
Polymicrogyria (PMG)	TUBA8	MT component		Abdollahi et al. (2009), Jaglin et al. (2009) and Poirier et al. (2010)
	TUBB2B	MT component	ID, epilepsy	Abdollahi et al. (2009), Jaglin et al. (2009), Jaglin and Chelly (2009), Poirier et al. (2010), Cushion et al. (2013) and Stouffer et al. (2016)
	TUBB3	MT component		Abdollahi et al. (2009), Jaglin et al. (2009), Poirier et al. (2010), Tischfield et al. (2010) and Whitman et al. (2016)
	KIF5C	Kinesin; MT-dependent motor	ID, seizures	Kanai et al. (2000), Homma et al. (2003), Poirier et al. (2013a) and Willemsen et al. (2014)
	KIF2A	Kinesin; MT-dependent motor	ID, epilepsy, developmental delay	Kanai et al. (2000), Homma et al. (2003) and Poirier et al. (2013a)
	DYNC1H1	Dynein; MT-dependent motor		Hafezparast et al. (2003), Shu et al. (2004), Ori-McKenney and Vallee (2011), Poirier et al. (2013a) and Zhao et al. (2016)
Lissencephaly	LIS1 (PAFAH1B1)	MT binding; dynein binding; MT stability; neuronal migration	ID, epilepsy	Reiner et al. (1993), Sapir et al. (1997), Hirotsune et al. (1998), Paylor et al. (1999), Smith et al. (2000), Fleck et al. (2000), Gambello et al. (2003), Shu et al. (2004), Li et al. (2005), Rehberg et al. (2005), Tsai et al. (2007), Bi et al. (2009), Youn et al. (2009), Egan et al. (2012), Moughamian et al. (2013), Vagnoni et al. (2016) and DeSantis et al. (2017)
	DCX	MT stability; promotes growth cone formation; neuronal migration	ID, epilepsy	Gleeson et al. (1998), des Portes et al. (1998), Pilz et al. (1998), Burgess and Reiner (2000), Corbo et al. (2002), Bai et al. (2003), Moores et al. (2004), Deuel et al. (2006), Tanaka et al. (2006), Bechstedt and Brouhard (2012) and Stouffer et al. (2016)
	TUBA1A	MT component	ID, PMG, epilepsy, motor delay	Poirier et al. (2007), Keays et al. (2007), Abdollahi et al. (2009), Jaglin et al. (2009), Jaglin and Chelly (2009), Poirier et al. (2010), Cushion et al. (2013), Fry et al. (2014), Bahi-Buisson et al. (2014) and Stouffer et al. (2016)⊥rule

### Type I Lissencephaly

One of the most widely known neuronal migration disorders is type I lissencephaly (“smooth brain”), also known as classic lissencephaly. This malformation is characterized by a spectrum of cortical abnormalities, which are defined by the absence or reduction of cortical folds (gyri) and grooves (sulci), making parts of the surface of the brain appear smooth. More severe forms of type I lissencephaly result in the complete loss of cortical folds (agyria), while milder forms result in the reduction of cortical folds (pachygyria), or areas of heterotopic bands of gray matter within the cortex (subcortical band heterotopia (SBH); Forman et al., 2005; Barkovich et al., 2012). Agyria and pachygyria arise from post-mitotic neurons failing to reach their proper positions, leading to a disorganized and thickened four-layer cortex instead of the normal six-layered cortex. In SBH, neurons migrate abnormally and form an extra layer of cells underneath the gray matter within the cortex. Type I lissencephaly is typically diagnosed in children within the first few months of life and patients often have many other symptoms including epilepsy, ID, developmental delays and motor function impairments.

Over the last few decades, studies of families with one or multiple individuals affected by type I lissencephaly led to the discovery of several mutations associated with MT-related genes (Kerjan and Gleeson, 2007). The first two genes to be identified were lissencephaly 1 (LIS1; Reiner et al., 1993), located on the short arm of chromosome 17, and doublecortin (DCX; Gleeson et al., 1998; des Portes et al., 1998), located on the X-chromosome. Human mutations of LIS1 (also known as PAFAH1B1, platelet-activating factor acetylhydrolase 1b, regulatory subunit 1) are heterozygous, affecting both males and females, while mutations of DCX are hemizygous, occurring on the X-chromosome, and largely affect only males. Mutations within LIS1 tend to be associated with a more severe phenotype in posterior brain regions, whereas mutations within DCX have the reverse effect with a more severe phenotype in frontal brain regions (Pilz et al., 1998). Both of these genes account for most cases of type I lissencephaly and code for MAPs that are necessary for neuronal differentiation and migration due to their essential interactions with the MT cytoskeleton (Pilz et al., 1998; Stouffer et al., 2016).

In migrating neurons, LIS1 predominately localizes to the centrosome where it aids in nuclear movement and acts as an adaptor protein to stabilize MTs by minimizing catastrophe (Sapir et al., 1997; Shu et al., 2004). LIS1 has also been shown to regulate the MT minus end directed motor, dynein (Smith et al., 2000; Rehberg et al., 2005), by increasing its localization and stabilization at MT plus ends (Li et al., 2005; DeSantis et al., 2017), and assisting in its transport of cargo along the MT (Egan et al., 2012; Moughamian et al., 2013; Vagnoni et al., 2016). DCX has been shown to be highly expressed in the growing axon, where it directly nucleates and stabilizes MTs by supporting their 13 protofilament structure and participates in promoting growth cone formation (Burgess and Reiner, 2000; Tint et al., 2009; Bechstedt and Brouhard, 2012; Jean et al., 2012). The interactions of LIS1 and DCX with MTs are regulated by numerous phosphorylation events (Sapir et al., 1999; Schaar et al., 2004). However, the specific sites of phosphorylation, as well as the kinases and phosphatases which regulate this activity, have still not been fully elucidated in relation to classic lissencephaly. It is possible that genetic mutations at specific phosphorylation sites may alter the interaction between LIS1 and DCX with the MT cytoskeleton, thereby changing their functions and producing variable phenotypic outcomes.

The creation of several mouse models of classic lissencephaly have been instrumental in investigating the cellular and molecular irregularities that occur due to the implicated genetic mutations of this disorder. Homozygous *Lis1* knockout (KO) mice die prenatally, indicating that LIS1 is absolutely essential for normal embryonic development (Hirotsune et al., 1998). Lis1-null heterozygous mice display motor coordination and cognition deficits, as well as severe disorganization of the cortical layers, hippocampus, and other areas within the brain (Paylor et al., 1999; Fleck et al., 2000; Youn et al., 2009). Moreover, overexpression of LIS1 caused severe structural brain abnormalities, including smaller brain size, distorted cellular organization and an increase in apoptotic cells, suggesting proper function of LIS1 is required in a dose-dependent manner (Bi et al., 2009). Inhibition of dynein results in a similar phenotype, further supporting the role of LIS1 in dynein function (Shu et al., 2004; Grabham et al., 2007; Tsai et al., 2007). Additional studies using histological analysis and BrdU labeling experiments have also provided strong evidence towards *in vivo* migrational defects in mice with reduced *Lis1* dosage, demonstrating that LIS1 is necessary for proper neuronal migration (Hirotsune et al., 1998; Gambello et al., 2003). Interestingly, mice with mutations in *Dcx* only display mild disordered neuronal migration phenotypes, mostly occurring in the hippocampus of both heterozygous females and hemizygous males (Corbo et al., 2002). In contrast, RNAi-directed depletion of Dcx showed abnormal radial migration of neurons in rat neocortex (Bai et al., 2003). It is possible that this variability of phenotypic severity may be due to compensation by other members of the DCX superfamily. For example, doublecortin-like kinase-1 (DCLK1) is a closely related gene to DCX, and *Dcx/Dckl1* KO mice display a more severe phenotype of cortical development, supporting the idea of genetic redundancy (Deuel et al., 2006; Tanaka et al., 2006). Together, *in vivo* and *in vitro* studies have confirmed that these two proteins are critical regulators of proper neuronal migration by working in combination, potentially in similar molecular pathways, to stabilize the MT cytoskeleton throughout neurodevelopment.

More recently, another genetic mutation linked to classic lissencephaly has been identified, occurring in the TUBA1A gene locus (Poirier et al., 2007). TUBA1A is an α-tubulin isotype specifically expressed in the developing nervous system and is required for proper MT structure and function (Keays et al., 2007; Poirier et al., 2007; Fry et al., 2014). Mice with heterozygous mutations of TubA1A were shown to have abnormal neuronal migration and lamination defects similar to the human phenotype (Keays et al., 2007, 2010). Subsequent patient studies revealed several TUBA1A mutations in locations that are predicted to interfere with the interactions between known binding partners, like DCX and other tubulins, suggesting that this may be a possible mechanism to explain how the mutations lead to cortical migration defects (Poirier et al., 2007; Bahi-Buisson et al., 2014), however, the molecular basis of these alterations still remains unclear. In addition to causing symptoms associated with classic lissencephaly, mutations within this gene have also been shown to cause a wide range of other cortical malformations, which will be discussed further in subsequent sections.

### Polymicrogyria

Another well-known neuronal migration disorder is Polymicrogyria (PMG), which is a spectrum of disorders characterized by excessive cerebral cortex folding and malformations of cortical layering. It has been described that hypoxia, congenital infections, inflammation of the microvasculature, as well as mitochondrial diseases are among the non-genetic causes that may lead to the cortical abnormalities related to PMG during early embryonic development (Gressens, 2000; Stutterd and Leventer, 2014). Clinical manifestations of PMG are heterogeneous and cause a wide range of developmental disabilities, making a uniform classification of this disorder difficult. Both environmental and genetic causes have been implicated in PMG; however, our current understanding of this cortical malformation still remains incomplete. Defining features of PMG are controversial, as some sources debate whether this disorder is truly due to a neuronal migration defect or a post-migrational defect, with abnormalities occurring after neurons are properly positioned to form the cortical layers (Judkins et al., 2011; Barkovich et al., 2012). These ambiguities make the need for more pathological and molecular studies essential in order to determine the mechanisms that lead to this developmental disorder.

Genetic studies have implicated numerous candidate genes associated with PMG, including transcription factors, signaling molecules and various cytoskeletal components, such as multiple tubulin isotypes and kinesin family members. The α-tubulin genes, TUBA1A and TUBA8, as well as the β-tubulin genes, TUBB2B and TUBB3, have all been identified in connection with PMG (Abdollahi et al., 2009; Jaglin et al., 2009; Poirier et al., 2010, 2013b). Mutations within these neuronally-expressed genes occur in a heterozygous fashion, with the exception of TUBA8 mutations, which are homozygous. Alterations within any of these genes can lead to structural and functional defects of the MT cytoskeleton, as well as interfere with the interactions between the MT cytoskeleton and other MT-related proteins. The abnormalities which arise from mutations of these tubulin isotypes are subtle yet distinct, indicating that they each play a unique role in regulating the MT cytoskeleton. For example, PMG patients harboring a mutation within TUBA8 have been shown to also have optic nerve hypoplasia and callosal dysgenesis (Abdollahi et al., 2009), whereas patients with a mutation in TUBB2B have asymmetrical PMG, dysmorphic basal ganglia, as well as heterotopic neuronal cells in the white matter areas of the cortex (Jaglin et al., 2009; Cushion et al., 2013). Additionally, axonal defects are present in all known TUBB3 mutations, which create abnormalities in axon targeting of the oculomotor muscles, leading to eye movement disorders such as congenital fibrosis of the external ocular muscles (CFEOM; Tischfield et al., 2010; Whitman et al., 2016). PMG patients with either TUBA1A or TUBB2B mutations have been found to display overlapping cortical malformations, possibly due to their similar roles in the formation of tubulin heterodimers, suggesting that MT stability may underlie some of the clinical phenotypes (Jaglin and Chelly, 2009; Cushion et al., 2013; Stouffer et al., 2016).

Within the last several years, studies have identified additional PMG-associated mutations within genes encoding several MT motor proteins including kinesin family members KIF5C and KIF2A, and a dynein-associated protein, DYNC1H1 (Poirier et al., 2013a; Fiorillo et al., 2014). Each mutation results in varying phenotypes, however, all of these genes play pivotal roles in regulating the MT cytoskeleton within neurons. KIF5C and KIF2A encode members of the kinesin superfamily, both of which are highly expressed in the developing nervous system and are involved in the intracellular transport of cargo along MTs (Kanai et al., 2000; Homma et al., 2003). *Kif2a* KO mice die shortly after birth and display numerous brain abnormalities including aberrant axon outgrowth and collateral branching, as well as delayed neuronal migration (Homma et al., 2003). Additionally, the MT-depolymerizing activity in neuronal growth cones was found to be reduced, indicating that KIF2A mechanistically regulates the growth cone via its interactions with the MT cytoskeleton during axon outgrowth. In contrast, Kif5C KO mice are viable and do not display any gross malformations within the CNS, aside from smaller brain size and a reduction of motor neurons (Kanai et al., 2000). This drastic difference in phenotype could be due to compensation by other closely related genes, suggesting that there may be functional redundancy among the kinesin family members. DYNC1H1 is a large subunit of the cytoplasmic dynein complex and various mouse models indicate that this gene is also vital for cortical development. Inactivation of the mouse homolog causes embryonic lethality, and N-ethyl-N-nitrosourea (ENU)-induced heterozygous missense mutations result in neurodegenerative diseases, as well as abnormal neuronal migration and retrograde axonal transport (Hafezparast et al., 2003; Ori-McKenney and Vallee, 2011; Zhao et al., 2016). Moreover, RNAi-directed reduction of *Dync1h1* similarly resulted in impaired neuronal migration (Shu et al., 2004). Taken together, these results highlight the importance of MT-dependent intracellular trafficking during early neural development.

### Microcephaly

Primary microcephaly (MCPH) is a neurodevelopmental disorder characterized by a smaller-than-normal head size arising from abnormal prenatal brain growth. This reduced head size occurs due to insufficient proliferation or increased apoptosis of neural stem cells, leading to a reduction in the number of neurons and impaired neurogenesis during development (Barkovich et al., 2012). Often, individuals with this disorder also present with ID, poor motor function, abnormal craniofacial features and seizures. A wide range of factors have been linked to cases of microcephaly such as genetic mutations, chromosomal abnormalities, vertically transmitted infections and other environmental factors (Barkovich et al., 2012).

Several genes have been discovered to be associated with MCPH, many of which code for proteins that localize to the centrosome and play a significant role in regulating MT dynamics. Mutations in abnormal spindle-like microcephaly-associated (ASPM) have been found to be the most common cause of MCPH (Duerinckx and Abramowicz, 2018). ASPM is essential for normal function and organization of mitotic spindle poles specifically within the developing brain (Kouprina et al., 2005). A recent study showed that ASPM can also recruit Katanin to promote MT severing and disassembly, suggesting that misregulation of this process may lead to microcephaly (Jiang et al., 2017). Likewise, microcephalin 1 (MCPH1), a gene which regulates DNA-damage responses, has also been found to be crucial for proper mitotic spindle alignment within neuroprogenitors (Gruber et al., 2011). Reduction of MCPH1 results in an imbalance between mitosis and the centrosome cycle, causing asymmetric cell division and dysregulation of chromosome condensation (Trimborn et al., 2004; Gruber et al., 2011). Another gene, SCL-TAL1 interrupting locus (STIL), encodes a cytoplasmic centriole duplication factor that is required for centriole formation and proper spindle positioning during embryonic brain development (Kumar et al., 2009; Kitagawa et al., 2011). Depletion of STIL results in a loss of centriole amplification, whereas overexpression leads to excess centriole formation (Vulprecht et al., 2012). CDK5 regulatory subunit associated protein 2 (CDK5RAP2) also codes for a protein that localizes to the centrosome and is involved in centrosome function and MT nucleation (Bond et al., 2005; Choi et al., 2010). Mutations of this gene result in cells displaying mitotic delay with abnormal spindle pole number and orientation (Lizarraga et al., 2010). Centromere protein J (CENPJ) is also involved in maintaining centrosome integrity and normal spindle pole morphology. Downregulation or loss of CENPJ leads to centrosome duplication abnormalities which contribute to spindle orientation defects, as well as improper neuronal migration and morphology (Kitagawa et al., 2011; Garcez et al., 2015). Together, these genes code for proteins which all play pivotal roles in regulating centrosome and spindle pole related functions, as well as MT dynamics. Disruptions within any of these genes may lead to defects in the cell cycle, MT nucleation and reduced proliferation of neurons, thus leading to microcephaly. The overlapping functions of these genes in relation to the centrosome are striking and provides strong evidence towards a link between proper centrosome function and MT dynamics during neurogenesis.

More recently, two other genes, PRUNE1 and KIF20B, have been identified in relation to MCPH. Prune exopolyphosphatase 1 (PRUNE1) encodes a member of the DHH (Asp-His-His) phosphoesterase protein superfamily important for cell motility. Genetic studies conducted by Zollo et al. (2017) identified biallelic mutations of PRUNE1 in 13 different individuals with microcephaly and developmental delay. Mutations in PRUNE1 impaired MT polymerization, cell migration and proliferation, suggesting that PRUNE1 may have a fundamental role in regulating these processes throughout cortical development. Kinesin Family Member 20B (KIF20B) is a MT plus end-directed motor that is required for the completion of cytokinesis and regulates cell polarity in neurons (McNeely et al., 2017). Loss of Kif20b disrupts cerebral cortex growth and cell polarization, as well as neurite outgrowth and branching (McNeely et al., 2017). Authors suggest that KIF20B may act to stabilize or bundle MTs in neurites to allow for proper polarization and outgrowth during brain development (McNeely et al., 2017).

## Microtubule-Associated Genes Linked to Intellectual Disabilities and Autism Spectrum Disorders

### Intellectual Disabilities

ID are complex neurodevelopmental disorders that affect a significant portion of the general population and are an immense health issue. ID is defined by an IQ score under 70 and is characterized by impaired intellectual and adaptive functioning that affects everyday living. ID may occur in isolation or it can be accompanied with other medical or behavioral symptoms such as seizures, craniofacial abnormalities, and microcephaly. ID can be caused by both genetic and environmental factors, with genetic causes representing up to 50% of all ID cases (Kaufman et al., 2010). Single gene mutations, as well as pathogenic copy number variants (CNVs), have been associated with ID, several of the implicated genes are involved in MT function.

Whole exome sequencing and next generation sequencing (NGS) studies have identified various mutations within kinesin superfamily members linked to ID. Willemsen et al. (2014) discovered pathogenic mutations in KIF4A and KIF5C in individuals from two different families. *In vivo* studies further confirmed the link between these genes and ID. Knockdown of both KIF4A and KIF5C disrupted the balance between excitatory and inhibitory synaptic activity, which may be a contributing factor that leads to ID (Willemsen et al., 2014). Furthermore, *de novo* mutations in KIF1A were found in several patients that had a range of cognitive and motor defects, including ID (Lee et al., 2015; Ohba et al., 2015). KIF1A, an anterograde motor protein, transports membranous organelles along MTs within axons and dendrites, and has been shown to be involved in synaptogenesis, as well as learning and memory (Kondo et al., 2012; McVicker et al., 2016). Reduction or loss of this protein results in abnormal interactions with MTs and disruptions in axonal and dendritic transport. Together, these results highlight the importance of the kinesin superfamily members during neural development and how alterations of their MT-dependent functions can lead to a spectrum of neurological defects.

A recent NGS study of large consanguineous Iranian families affected by ID identified a novel mutation in CAP-Gly domain containing linker protein 1 (CLIP1), which encodes a +TIP, CLIP-170, that localizes to the ends of growing MTs (Larti et al., 2015). CLIP1 regulates MT behavior and participates in MT-mediated transport in neurons (Jaworski et al., 2008; Swiech et al., 2011). The protein encoded by CLIP1 was absent from cell lines derived from these ID patients, suggesting that loss of CLIP1 function can lead to cognitive impairments. It has also been shown that CLIP-170 may interact with LIS1 to mediate the recruitment of dynein to MTs and regulate MT dynamics (Coquelle et al., 2002). It is possible that the interaction between CLIP1 and LIS1 may be important for proper neuronal migration during brain development.

Microdeletions on chromosome 13 encompassing several genes, including katanin catalytic subunit A1 like 1 (KATNAL1), were found to result in ID and microcephaly (Bartholdi et al., 2014). KATNAL1 encodes a MT-severing enzyme that regulates the remodeling of cellular MT arrays. Mouse lines carrying a loss-of-function allele in *Katnal1* display defects in learning and memory, as well as abnormal neuronal migration and morphology (Banks et al., 2018). However, there are genetic discrepancies between humans and mice. Human patients are heterozygous for the KATNAL1 deletion, whereas heterozygous mice show no overt phenotypes, suggesting that further investigations are required to understand the causative mechanisms in regards to this genetic mutation (Bartholdi et al., 2014; Banks et al., 2018). Nevertheless, it is evident that KATNAL1 plays an important role during several neuronal processes, and that perturbations of KATNAL1 function can lead to various defects which may eventually contribute to neurodevelopmental disorders.

X-linked ID have been associated with mutations in midline 2 (MID2), a gene that codes for an ubiquitin ligase, which localizes to MTs and regulates their activity during neural tube closure (Geetha et al., 2014). Mid2 was shown to localize and ubiquitinate Astrin, which is a MT organizing protein that regulates MTs during cell division. Loss of Mid2 led to the stabilization of Astrin, causing defects in MT organization, cytokinesis and cell death (Gholkar et al., 2016). This suggests that ubiquitination of Astrin by Mid2 is essential for regulating MT function during cell division and could explain how mutations of MID2 lead to X-linked ID.

### Autism Spectrum Disorders

ASD are a heterogeneous group of disorders characterized by a wide range of symptoms and disabilities that can vary in severity. These symptoms include verbal and non-verbal communication deficits, difficulties with social interactions, repetitive behaviors and restrictive interests. Individuals with ASD can also present with additional medical conditions such as epilepsy, motor function impairments, ID, anxiety and sleep disorders. The pathogenesis of ASD is not yet fully understood and a majority of ASD cases do not have a specific known cause. However, increased research efforts have identified various genetic mutations which have been linked to ASD, including several MT-associated genes (Pinto et al., 2014).

One of these genes is autism susceptibility candidate 2 (AUTS2), however the exact functions of AUTS2 have not yet been entirely characterized, though studies suggest that it may play a significant role during early brain development (Kalscheuer et al., 2007). AUTS2 has been found to be highly expressed in the developing brain of zebrafish, and knockdown of this gene resulted in microcephaly along with a reduction in the total number of neurons (Oksenberg et al., 2013). Further studies found that the protein functioned as a regulator of Rac1, a Rho-family GTPase that is crucial for coordinating cytoskeletal rearrangements (Hori et al., 2014). The reduction or loss of Auts2 in mice caused abnormal morphologies of embryonic neurons and impaired their migration (Hori et al., 2014). Knockdown of Auts2 also suppressed the activation of c-Jun N-terminal kinase (JNK). JNK is regulated by Rac1 and is involved in MT formation, as well as MT dynamics at leading processes of migrating neurons (Kawauchi et al., 2003). Taken together, alterations in AUTS2 may inhibit its regulation of Rac1, which then has downstream effects on subsequent target molecules, like JNK, that can lead to defects in cytoskeletal remodeling in migrating neurons.

Another gene implicated in ASD is activity-dependent neuroprotective protein (ADNP; Helsmoortel et al., 2014). This gene is part of the SWI/SNF chromatin remodeling complex and is vital for brain development (Mandel et al., 2007; Helsmoortel et al., 2014). *Adnp*−/− mice die prenatally due to failure of neural tube closure, and *Adnp+/−* mice have increased neuronal death along with abnormal cognitive functioning (Vulih-Shultzman et al., 2007). ADNP was found to be associated with tau mRNA splicing (Schirer et al., 2014), and also participates in the recruitment of Tau to MTs, possibly to prevent free Tau accumulation that eventually leads to neurodegenerative disorders (Oz et al., 2012). Furthermore, ADNP has been shown to directly interact with MT EBs, EB1 and EB3, to promote neurite outgrowth and dendritic spine formation (Oz et al., 2014; Ivashko-Pachima et al., 2017). Together, these findings indicate that mutations of ADNP may alter its interactions with several MT-associated proteins, which have negative downstream effects that alter MT dynamics and hinder different neuronal processes during early development.

Dysregulation of several MT-associated kinases have also been linked to ASD. Janus kinase and MT interacting protein 1 (JAKMIP1) is an RNA binding protein that is highly expressed in glutamatergic neurons (Couve et al., 2004), and has been shown to modify MT polymers and influence MT dynamics (Steindler et al., 2004). Rare deletions of this gene have been found in individuals with ASD (Kaminsky et al., 2011; Hedges et al., 2012; Poultney et al., 2013), and loss of *Jakmip1* in mice results in autistic-like behavior, possibly by affecting the expression of downstream mRNA targets during synaptogenesis (Berg et al., 2015). It has also been suggested that JAKMIP1 modulates intracellular trafficking of GABA receptors via its interaction with the MT cytoskeleton (Vidal et al., 2007). Given these results, it is possible that mutations of JAKMIP1 cause defects in synapse formation, specifically within glutamatergic neurons, that may eventually lead to ASD. Another MT-associated kinase, MT affinity regulating kinase 1 (MARK1), was shown to have altered transcript and protein levels in postmortem brains from patients with ASD (Maussion et al., 2008). MARK1 is a kinase which phosphorylates several MAPs, causing them to dissociate from MTs (Drewes et al., 1997). MARK1 also participates in the regulation of mitochondrial trafficking along MTs in both axons and dendrites and plays a significant role during neuronal polarization and migration (Mandelkow et al., 2004; Maussion et al., 2008). The reduction or overexpression of MARK1 has been shown to cause defects in synaptic function as well as cell migration (Maussion et al., 2008; Sapir et al., 2008). It is possible that mutations within MARK1 modulate its phosphorylation activity of MAPs, leading to aberrant alterations of MT dynamics that disrupt proper neural development.

## Conclusion

The elaborate MT cytoskeletal network plays many instrumental roles during development of the nervous system. Young neurons rely on the dynamic properties of MTs in order to proliferate and migrate to their final destinations. Moreover, MTs are vital for the formation and extension of both axons and dendrites, which enables the neuron to navigate through the extracellular terrain to create synapses and connections with neighboring cells. Elucidating the molecular mechanisms responsible for regulating the MT cytoskeleton throughout specific stages of neural development still remain an important area of research. In recent years, genetic studies have uncovered numerous mutations within genes that negatively affect the MT cytoskeleton, causing abnormalities in neural migration, proliferation and connectivity. The discovery of mutations occurring within MT-associated genes that lead to neurodevelopmental disorders has provided an opportunity to investigate the functions of MTs at the cellular and molecular level during brain development. Future studies must continue to focus on understanding how these mutations contribute to altered MT function underlying various brain malformations, which will allow insight into the essential roles MTs and their associated proteins play during neural development.

## Author Contributions

ML and LAL conceived of the manuscript. ML, JT and LAL wrote and edited the manuscript.

## Conflict of Interest Statement

The authors declare that the research was conducted in the absence of any commercial or financial relationships that could be construed as a potential conflict of interest.

## References

[B1] Abdollahi M. R. Morrison E. Sirey T. Molnár Z. Hayward B. E. Carr I. M.. (2009). Mutation of the variant α-tubulin TUBA8 results in polymicrogyria with optic nerve hypoplasia. Am. J. Hum. Genet. 85, 737–744. 10.1016/j.ajhg.2009.10.007 19896110 PMC2775839

[B2] Akhmanova A. Steinmetz M. O. (2008). Tracking the ends: a dynamic protein network controls the fate of microtubule tips. Nat. Rev. Mol. Cell Biol. 9, 309–322. 10.1038/nrm2369 18322465

[B3] Alves-Silva J. Sánchez-Soriano N. Beaven R. Klein M. Parkin J. Millard T. H.. (2012). Spectraplakins promote microtubule-mediated axonal growth by functioning as structural microtubule-associated proteins and EB1-dependent +TIPs (tip interacting proteins). J. Neurosci. 32, 9143–9158. 10.1523/JNEUROSCI.0416-12.2012 22764224 PMC3666083

[B4] Armijo-Weingart L. Gallo G. (2017). It takes a village to raise a branch: cellular mechanisms of the initiation of axon collateral branches. Mol. Cell. Neurosci. 84, 36–47. 10.1016/j.mcn.2017.03.007 28359843 PMC5617777

[B5] Bahi-Buisson N. Poirier K. Fourniol F. Saillour Y. Valence S. Lebrun N.. (2014). The wide spectrum of tubulinopathies: what are the key features for the diagnosis? Brain 137, 1676–1700. 10.1093/brain/awu082 24860126

[B6] Bai J. Ramos R. L. Ackman J. B. Thomas A. M. Lee R. V. LoTurco J. J. (2003). RNAi reveals doublecortin is required for radial migration in rat neocortex. Nat. Neurosci. 6, 1277–1283. 10.1038/nn1153 14625554

[B7] Banks G. Lassi G. Hoerder-Suabedissen A. Tinarelli F. Simon M. M. Wilcox A.. (2018). A missense mutation in Katnal1 underlies behavioural, neurological and ciliary anomalies. Mol. Psychiatry 23, 713–722. 10.1038/mp.2017.54 28373692 PMC5761721

[B8] Barkovich A. J. Guerrini R. Kuzniecky R. I. Jackson G. D. Dobyns W. B. (2012). A developmental and genetic classification for malformations of cortical development: update 2012. Brain 135, 1348–1369. 10.1093/brain/aws019 22427329 PMC3338922

[B9] Bartholdi D. Stray-Pedersen A. Azzarello-Burri S. Kibaek M. Kirchhoff M. Oneda B.. (2014). A newly recognized 13q12.3 microdeletion syndrome characterized by intellectual disability, microcephaly and eczema/atopic dermatitis encompassing the HMGB1 and KATNAL1 genes. Am. J. Med. Genet. A 164A, 1277–1283. 10.1002/ajmg.a.36439 24664804

[B10] Bearce E. A. Erdogan B. Lowery L. A. (2015). TIPsy tour guides: how microtubule plus-end tracking proteins (+TIPs) facilitate axon guidance. Front. Cell. Neurosci. 9:241. 10.3389/fncel.2015.00241 26175669 PMC4485311

[B11] Bechstedt S. Brouhard G. J. (2012). Doublecortin recognizes the 13-protofilament microtubule cooperatively and tracks microtubule ends. Dev. Cell 23, 181–192. 10.1016/j.devcel.2012.05.006 22727374 PMC3951992

[B12] Berg J. M. Lee C. Chen L. Galvan L. Cepeda C. Chen J. Y.. (2015). JAKMIP1, a novel regulator of neuronal translation, modulates synaptic function and autistic-like behaviors in mouse. Neuron 88, 1173–1191. 10.1016/j.neuron.2015.10.031 26627310 PMC4829343

[B13] Bi W. Sapir T. Shchelochkov O. A. Zhang F. Withers M. A. Hunter J. V.. (2009). Increased LIS1 expression affects human and mouse brain development. Nat. Genet. 41, 168–177. 10.1038/ng.302 19136950 PMC4396744

[B14] Biswas S. Kalil K. (2018). The microtubule-associated protein tau mediates the organization of microtubules and their dynamic exploration of actin-rich lamellipodia and filopodia of cortical growth cones. J. Neurosci. 38, 291–307. 10.1523/JNEUROSCI.2281-17.2017 29167405 PMC5761611

[B15] Bodaleo F. J. Montenegro-Venegas C. Henríquez D. R. Court F. A. González-Billault C. (2016). Microtubule-associated protein 1B (MAP1B)-deficient neurons show structural presynaptic deficiencies *in vitro* and altered presynaptic physiology. Sci. Rep. 6:30069. 10.1038/srep32275 27425640 PMC4948024

[B16] Bond J. Roberts E. Springell K. Lizarraga S. B. Lizarraga S. Scott S.. (2005). A centrosomal mechanism involving CDK5RAP2 and CENPJ controls brain size. Nat. Genet. 37, 353–355. 10.1038/ng1539 15793586

[B17] Bouquet C. Soares S. von Boxberg Y. Ravaille-Veron M. Propst F. Nothias F. (2004). Microtubule-associated protein 1B controls directionality of growth cone migration and axonal branching in regeneration of adult dorsal root ganglia neurons. J. Neurosci. 24, 7204–7213. 10.1523/JNEUROSCI.2254-04.2004 15306655 PMC6729172

[B18] Burgess H. A. Reiner O. (2000). Doublecortin-like kinase is associated with microtubules in neuronal growth cones. Mol. Cell. Neurosci. 16, 529–541. 10.1006/mcne.2000.0891 11083916

[B19] Cammarata G. M. Bearce E. A. Lowery L. A. (2016). Cytoskeletal social networking in the growth cone: how +TIPs mediate microtubule-actin cross-linking to drive axon outgrowth and guidance. Cytoskeleton 73, 461–476. 10.1002/cm.21272 26783725 PMC4955630

[B20] Chakraborti S. Natarajan K. Curiel J. Janke C. Liu J. (2016). The emerging role of the tubulin code: from the tubulin molecule to neuronal function and disease. Cytoskeleton 73, 521–550. 10.1002/cm.21290 26934450

[B21] Choi Y.-K. Liu P. Sze S. K. Dai C. Qi R. Z. (2010). CDK5RAP2 stimulates microtubule nucleation by the γ-tubulin ring complex. J. Cell Biol. 191, 1089–1095. 10.1083/jcb.201007030 21135143 PMC3002024

[B22] Conde C. Cáceres A. (2009). Microtubule assembly, organization and dynamics in axons and dendrites. Nat. Rev. Neurosci. 10, 319–332. 10.1038/nrn2631 19377501

[B23] Coquelle F. M. Caspi M. Cordelières F. P. Dompierre J. P. Dujardin D. L. Koifman C.. (2002). LIS1, CLIP-170’s key to the dynein/dynactin pathway. Mol. Cell. Biol. 22, 3089–3102. 10.1128/mcb.22.9.3089-3102.2002 11940666 PMC133759

[B24] Corbo J. C. Deuel T. A. Long J. M. LaPorte P. Tsai E. Wynshaw-Boris A.. (2002). Doublecortin is required in mice for lamination of the hippocampus but not the neocortex. J. Neurosci. 22, 7548–7557. 10.1523/JNEUROSCI.22-17-07548.2002 12196578 PMC6757990

[B25] Couve A. Restituito S. Brandon J. M. Charles K. J. Bawagan H. Freeman K. B.. (2004). Marlin-1, a novel RNA-binding protein associates with GABA receptors. J. Biol. Chem. 279, 13934–13943. 10.1074/jbc.M311737200 14718537

[B26] Cox G. A. Mahaffey C. L. Nystuen A. Letts V. A. Frankel W. N. (2000). The mouse fidgetin gene defines a new role for AAA family proteins in mammalian development. Nat. Genet. 26, 198–202. 10.1038/79923 11017077

[B27] Cushion T. D. Dobyns W. B. Mullins J. G. L. Stoodley N. Chung S.-K. Fry A. E.. (2013). Overlapping cortical malformations and mutations in TUBB2B and TUBA1A. Brain 136, 536–548. 10.1093/brain/aws338 23361065

[B28] Dehmelt L. Halpain S. (2005). The MAP2/Tau family of microtubule-associated proteins. Genome Biol. 6:204. 10.1186/gb-2004-6-1-204 15642108 PMC549057

[B29] Dehmelt L. Nalbant P. Steffen W. Halpain S. (2006). A microtubule-based, dynein-dependent force induces local cell protrusions: implications for neurite initiation. Brain Cell Biol. 35, 39–56. 10.1007/s11068-006-9001-0 17940912

[B30] Dent E. W. (2017). Of microtubules and memory: implications for microtubule dynamics in dendrites and spines. Mol. Biol. Cell 28, 1–8. 10.1091/mbc.E15-11-0769 28035040 PMC5221613

[B32] Dent E. W. Callaway J. L. Szebenyi G. Baas P. W. Kalil K. (1999). Reorganization and movement of microtubules in axonal growth cones and developing interstitial branches. J. Neurosci. 19, 8894–8908. 10.1523/JNEUROSCI.19-20-08894.1999 10516309 PMC6782770

[B33] Dent E. W. Gupton S. L. Gertler F. B. (2011). The growth cone cytoskeleton in axon outgrowth and guidance. Cold Spring Harb. Perspect. Biol. 3:a001800. 10.1101/cshperspect.a001800 21106647 PMC3039926

[B31] Dent E. W. Kalil K. (2001). Axon branching requires interactions between dynamic microtubules and actin filaments. J. Neurosci. 21, 9757–9769. 10.1523/JNEUROSCI.21-24-09757.2001 11739584 PMC6763027

[B34] Dent E. W. Kwiatkowski A. V. Mebane L. M. Philippar U. Barzik M. Rubinson D. A.. (2007). Filopodia are required for cortical neurite initiation. Nat. Cell Biol. 9, 1347–1359. 10.1038/ncb1654 18026093

[B35] DeSantis M. E. Cianfrocco M. A. Htet Z. M. Tran P. T. Reck-Peterson S. L. Leschziner A. E. (2017). Lis1 has two opposing modes of regulating cytoplasmic dynein. Cell 170, 1197.e12–1208.e12. 10.1016/j.cell.2017.08.037 28886386 PMC5625841

[B154] des Portes V. Francis F. Pinard J. M. Desguerre I. Moutard M. L. Snoeck I.. (1998). Doublecortin is the major gene causing X-linked subcortical laminar heterotopia (SCLH). Hum. Mol. Genet. 7, 1063–1070. 10.1093/hmg/7.7.1063 9618162

[B36] Deuel T. A. S. Liu J. S. Corbo J. C. Yoo S.-Y. Rorke-Adams L. B. Walsh C. A. (2006). Genetic interactions between doublecortin and doublecortin-like kinase in neuronal migration and axon outgrowth. Neuron 49, 41–53. 10.1016/j.neuron.2005.10.038 16387638

[B37] Drewes G. Ebneth A. Preuss U. Mandelkow E. M. Mandelkow E. (1997). MARK, a novel family of protein kinases that phosphorylate microtubule-associated proteins and trigger microtubule disruption. Cell 89, 297–308. 10.1016/s0092-8674(00)80208-1 9108484

[B38] Duerinckx S. Abramowicz M. (2018). The genetics of congenitally small brains. Semin. Cell Dev. Biol. 76, 76–85. 10.1016/j.semcdb.2017.09.015 28912110

[B39] Egan M. J. Tan K. Reck-Peterson S. L. (2012). Lis1 is an initiation factor for dynein-driven organelle transport. J. Cell Biol. 197, 971–982. 10.1083/jcb.201112101 22711696 PMC3384415

[B40] Eom T.-Y. Stanco A. Guo J. Wilkins G. Deslauriers D. Yan J.. (2014). Differential regulation of microtubule severing by APC underlies distinct patterns of projection neuron and interneuron migration. Dev. Cell 31, 677–689. 10.1016/j.devcel.2014.11.022 25535916 PMC4289145

[B41] Erdogan B. Cammarata G. M. Lee E. J. Pratt B. C. Francl A. F. Rutherford E. L.. (2017). The microtubule plus-end-tracking protein TACC3 promotes persistent axon outgrowth and mediates responses to axon guidance signals during development. Neural Dev. 12:3. 10.1186/s13064-017-0080-7 28202041 PMC5312526

[B42] Fiorillo C. Moro F. Yi J. Weil S. Brisca G. Astrea G.. (2014). Novel dynein DYNC1H1 neck and motor domain mutations link distal spinal muscular atrophy and abnormal cortical development. Hum. Mutat. 35, 298–302. 10.1002/humu.22491 24307404 PMC4109683

[B43] Fleck M. W. Hirotsune S. Gambello M. J. Phillips-Tansey E. Suares G. Mervis R. F.. (2000). Hippocampal abnormalities and enhanced excitability in a murine model of human lissencephaly. J. Neurosci. 20, 2439–2450. 10.1523/JNEUROSCI.20-07-02439.2000 10729324 PMC6772237

[B44] Forman M. S. Squier W. Dobyns W. B. Golden J. A. (2005). Genotypically defined lissencephalies show distinct pathologies. J. Neuropathol. Exp. Neurol. 64, 847–857. 10.1097/01.jnen.0000182978.56612.41 16215456

[B45] Fry A. E. Cushion T. D. Pilz D. T. (2014). The genetics of lissencephaly. Am. J. Med. Genet. C Semin Med. Genet. 166C, 198–210. 10.1002/ajmg.c.31402 24862549

[B46] Götz M. Huttner W. B. (2005). The cell biology of neurogenesis. Nat. Rev. Mol. Cell Biol. 6, 777–788. 10.1038/nrm1739 16314867

[B47] Gallo G. (2016). Coordination of the axonal cytoskeleton during the emergence of axon collateral branches. Neural Regen. Res. 11, 709–711. 10.4103/1673-5374.182684 27335541 PMC4904448

[B48] Gallo G. Letourneau P. C. (1999). Different contributions of microtubule dynamics and transport to the growth of axons and collateral sprouts. J. Neurosci. 19, 3860–3873. 10.1523/JNEUROSCI.19-10-03860.1999 10234018 PMC6782725

[B49] Gambello M. J. Darling D. L. Yingling J. Tanaka T. Gleeson J. G. Wynshaw-Boris A. (2003). Multiple dose-dependent effects of Lis1 on cerebral cortical development. J. Neurosci. 23, 1719–1729. 10.1523/JNEUROSCI.23-05-01719.2003 12629176 PMC6741979

[B50] Garcez P. P. Diaz-Alonso J. Crespo-Enriquez I. Castro D. Bell D. Guillemot F. (2015). Cenpj/CPAP regulates progenitor divisions and neuronal migration in the cerebral cortex downstream of Ascl1. Nat. Commun. 6:6474. 10.1038/ncomms7474 25753651 PMC4366522

[B51] Geetha T. S. Michealraj K. A. Kabra M. Kaur G. Juyal R. C. Thelma B. K. (2014). Targeted deep resequencing identifies MID2 mutation for X-linked intellectual disability with varied disease severity in a large kindred from India. Hum. Mutat. 35, 41–44. 10.1002/humu.22453 24115387

[B52] Gholkar A. A. Senese S. Lo Y.-C. Vides E. Contreras E. Hodara E.. (2016). The X-linked-intellectual-disability-associated ubiquitin ligase Mid2 interacts with astrin and regulates astrin levels to promote cell division. Cell Rep. 14, 180–188. 10.1016/j.celrep.2015.12.035 26748699 PMC4724641

[B53] Gleeson J. G. Allen K. M. Fox J. W. Lamperti E. D. Berkovic S. Scheffer I.. (1998). Doublecortin, a brain-specific gene mutated in human X-linked lissencephaly and double cortex syndrome, encodes a putative signaling protein. Cell 92, 63–72. 10.1016/s0092-8674(00)80899-5 9489700

[B54] Gozes I. Helsmoortel C. Vandeweyer G. Van der Aa N. Kooy F. Bedrosian-Sermone S. (2018). The compassionate side of neuroscience: tony sermone’s undiagnosed genetic journey—ADNP mutation. J. Mol. Neurosci. 56, 751–757. 10.1007/s12031-018-1028-z 26168855

[B55] Gozes I. Patterson M. C. Van Dijck A. Kooy R. F. Peeden J. N. Eichenberger J. A.. (2017a). The eight and a half year journey of undiagnosed AD: gene sequencing and funding of advanced genetic testing has led to hope and new beginnings. Front. Endocrinol. 8:107. 10.3389/fendo.2017.00107 28579975 PMC5437153

[B56] Gozes I. Van Dijck A. Hacohen-Kleiman G. Grigg I. Karmon G. Giladi E.. (2017b). Premature primary tooth eruption in cognitive/motor-delayed ADNP-mutated children. Transl. Psychiatry 7:e1043. 10.1038/tp.2017.128 28221363 PMC5438031

[B57] Grabham P. W. Seale G. E. Bennecib M. Goldberg D. J. Vallee R. B. (2007). Cytoplasmic dynein and LIS1 are required for microtubule advance during growth cone remodeling and fast axonal outgrowth. J. Neurosci. 27, 5823–5834. 10.1523/JNEUROSCI.1135-07.2007 17522326 PMC6672755

[B58] Gressens P. (2000). Mechanisms and disturbances of neuronal migration. Pediatr. Res. 48, 725–730. 10.1203/00006450-200012000-00004 11102537

[B59] Gruber R. Zhou Z. Sukchev M. Joerss T. Frappart P.-O. Wang Z.-Q. (2011). MCPH1 regulates the neuroprogenitor division mode by coupling the centrosomal cycle with mitotic entry through the Chk1-Cdc25 pathway. Nat. Cell Biol. 13, 1325–1334. 10.1038/ncb2342 21947081

[B60] Gu J. Firestein B. L. Zheng J. Q. (2008). Microtubules in dendritic spine development. J. Neurosci. 28, 12120–12124. 10.1523/JNEUROSCI.2509-08.2008 19005076 PMC2605155

[B61] Gumy L. F. Katrukha E. A. Grigoriev I. Jaarsma D. Kapitein L. C. Akhmanova A.. (2017). MAP2 defines a pre-axonal filtering zone to regulate KIF1- versus KIF5-dependent cargo transport in sensory neurons. Neuron 94, 347.e7–362.e7. 10.1016/j.neuron.2017.03.046 28426968

[B62] Hafezparast M. Klocke R. Ruhrberg C. Marquardt A. Ahmad-Annuar A. Bowen S.. (2003). Mutations in dynein link motor neuron degeneration to defects in retrograde transport. Science 300, 808–812. 10.1126/science.1083129 12730604

[B63] Harada A. Teng J. Takei Y. Oguchi K. Hirokawa N. (2002). MAP2 is required for dendrite elongation, PKA anchoring in dendrites, and proper PKA signal transduction. J. Cell Biol. 158, 541–549. 10.1083/jcb.200110134 12163474 PMC2173814

[B64] Hazan J. Fonknechten N. Mavel D. Paternotte C. Samson D. Artiguenave F.. (1999). Spastin, a new AAA protein, is altered in the most frequent form of autosomal dominant spastic paraplegia. Nat. Genet. 23, 296–303. 10.1038/15472 10610178

[B65] Hedges D. J. Hamilton-Nelson K. L. Sacharow S. J. Nations L. Beecham G. W. Kozhekbaeva Z. M.. (2012). Evidence of novel fine-scale structural variation at autism spectrum disorder candidate loci. Mol. Autism 3:2. 10.1186/2040-2392-3-2 22472195 PMC3352055

[B66] Helsmoortel C. Vulto-van Silfhout A. T. Coe B. P. Vandeweyer G. Rooms L. van den Ende J.. (2014). A SWI/SNF-related autism syndrome caused by *de novo* mutations in ADNP. Nat. Genet. 46, 380–384. 10.1038/ng.2899 24531329 PMC3990853

[B67] Hirokawa N. Niwa S. Tanaka Y. (2010). Molecular motors in neurons: transport mechanisms and roles in brain function, development and disease. Neuron 68, 610–638. 10.1016/j.neuron.2010.09.039 21092854

[B68] Hirotsune S. Fleck M. W. Gambello M. J. Bix G. J. Chen A. Clark G. D.. (1998). Graded reduction of Pafah1b1 (Lis1) activity results in neuronal migration defects and early embryonic lethality. Nat. Genet. 19, 333–339. 10.1038/1221 9697693

[B69] Homma N. Takei Y. Tanaka Y. Nakata T. Terada S. Kikkawa M.. (2003). Kinesin superfamily protein 2A (KIF2A) functions in suppression of collateral branch extension. Cell 114, 229–239. 10.1016/s0092-8674(03)00522-1 12887924

[B70] Hoogenraad C. C. Bradke F. (2009). Control of neuronal polarity and plasticity—a renaissance for microtubules? Trends Cell Biol. 19, 669–676. 10.1016/j.tcb.2009.08.006 19801190

[B71] Hori K. Nagai T. Shan W. Sakamoto A. Taya S. Hashimoto R.. (2014). Cytoskeletal regulation by AUTS2 in neuronal migration and neuritogenesis. Cell Rep. 9, 2166–2179. 10.1016/j.celrep.2014.11.045 25533347

[B72] Ivashko-Pachima Y. Sayas C. L. Malishkevich A. Gozes I. (2017). ADNP/NAP dramatically increase microtubule end-binding protein-Tau interaction: a novel avenue for protection against tauopathy. Mol. Psychiatry 22, 1335–1344. 10.1038/mp.2016.255 28115743

[B73] Jacobson C. Schnapp B. Banker G. A. (2006). A change in the selective translocation of the Kinesin-1 motor domain marks the initial specification of the axon. Neuron 49, 797–804. 10.1016/j.neuron.2006.02.005 16543128

[B74] Jaglin X. H. Chelly J. (2009). Tubulin-related cortical dysgeneses: microtubule dysfunction underlying neuronal migration defects. Trends Genet. 25, 555–566. 10.1016/j.tig.2009.10.003 19864038

[B75] Jaglin X. H. Poirier K. Saillour Y. Buhler E. Tian G. Bahi-Buisson N.. (2009). Mutations in the β-tubulin gene TUBB2B result in asymmetrical polymicrogyria. Nat. Genet. 41, 746–752. 10.1038/ng.380 19465910 PMC2883584

[B76] Janning D. Igaev M. Sündermann F. Brühmann J. Beutel O. Heinisch J. J.. (2014). Single-molecule tracking of tau reveals fast kiss-and-hop interaction with microtubules in living neurons. Mol. Biol. Cell 25, 3541–3551. 10.1091/mbc.E14-06-1099 25165145 PMC4230615

[B77] Jaworski J. Hoogenraad C. C. Akhmanova A. (2008). Microtubule plus-end tracking proteins in differentiated mammalian cells. Int. J. Biochem. Cell Biol. 40, 619–637. 10.1016/j.biocel.2007.10.015 18023603

[B78] Jaworski J. Kapitein L. C. Gouveia S. M. Dortland B. R. Wulf P. S. Grigoriev I.. (2009). Dynamic microtubules regulate dendritic spine morphology and synaptic plasticity. Neuron 61, 85–100. 10.1016/j.neuron.2008.11.013 19146815

[B79] Jayachandran P. Olmo V. N. Sanchez S. P. McFarland R. J. Vital E. Werner J. M.. (2016). Microtubule-associated protein 1b is required for shaping the neural tube. Neural Dev. 11:1. 10.1186/s13064-015-0056-4 26782621 PMC4717579

[B80] Jean D. C. Baas P. W. Black M. M. (2012). A novel role for doublecortin and doublecortin-like kinase in regulating growth cone microtubules. Hum. Mol. Genet. 21, 5511–5527. 10.1093/hmg/dds395 23001563 PMC3516135

[B81] Jiang K. Rezabkova L. Hua S. Liu Q. Capitani G. Altelaar A. F. M.. (2017). Microtubule minus-end regulation at spindle poles by an ASPM-katanin complex. Nat. Cell Biol. 19, 480–492. 10.1038/ncb3511 28436967 PMC5458804

[B82] Judkins A. R. Martinez D. Ferreira P. Dobyns W. B. Golden J. A. (2011). Polymicrogyria includes fusion of the molecular layer and decreased neuronal populations but normal cortical laminar organization. J. Neuropathol. Exp. Neurol. 70, 438–443. 10.1097/NEN.0b013e31821ccf1c 21572338 PMC3113653

[B83] Kahn O. I. Baas P. W. (2016). Microtubules and growth cones: motors drive the turn. Trends Neurosci. 39, 433–440. 10.1016/j.tins.2016.04.009 27233682 PMC4930683

[B84] Kalil K. Dent E. W. (2014). Branch management: mechanisms of axon branching in the developing vertebrate CNS. Nat. Rev. Neurosci. 15, 7–18. 10.1038/nrn3650 24356070 PMC4063290

[B85] Kalscheuer V. M. FitzPatrick D. Tommerup N. Bugge M. Niebuhr E. Neumann L. M.. (2007). Mutations in autism susceptibility candidate 2 (AUTS2) in patients with mental retardation. Hum. Genet. 121, 501–509. 10.1007/s00439-006-0284-0 17211639

[B86] Kaminsky E. B. Kaul V. Paschall J. Church D. M. Bunke B. Kunig D.. (2011). An evidence-based approach to establish the functional and clinical significance of copy number variants in intellectual and developmental disabilities. Genet. Med. 13, 777–784. 10.1097/GIM.0b013e31822c79f9 21844811 PMC3661946

[B87] Kanai Y. Okada Y. Tanaka Y. Harada A. Terada S. Hirokawa N. (2000). KIF5C, a novel neuronal kinesin enriched in motor neurons. J. Neurosci. 20, 6374–6384. 10.1523/JNEUROSCI.20-17-06374.2000 10964943 PMC6772948

[B88] Kapitein L. C. Hoogenraad C. C. (2011). Which way to go? Cytoskeletal organization and polarized transport in neurons. Mol. Cell. Neurosci. 46, 9–20. 10.1016/j.mcn.2010.08.015 20817096

[B89] Kapitein L. C. Hoogenraad C. C. (2015). Building the neuronal microtubule cytoskeleton. Neuron 87, 492–506. 10.1016/j.neuron.2015.05.046 26247859

[B90] Kapitein L. C. Schlager M. A. Kuijpers M. Wulf P. S. van Spronsen M. MacKintosh F. C.. (2010). Mixed microtubules steer dynein-driven cargo transport into dendrites. Curr. Biol. 20, 290–299. 10.1016/j.cub.2009.12.052 20137950

[B91] Karabay A. Yu W. Solowska J. M. Baird D. H. Baas P. W. (2004). Axonal growth is sensitive to the levels of katanin, a protein that severs microtubules. J. Neurosci. 24, 5778–5788. 10.1523/JNEUROSCI.1382-04.2004 15215300 PMC6729225

[B92] Kaufman L. Ayub M. Vincent J. B. (2010). The genetic basis of non-syndromic intellectual disability: a review. J. Neurodev. Disord. 2, 182–209. 10.1007/s11689-010-9055-2 21124998 PMC2974911

[B93] Kawauchi T. Chihama K. Nabeshima Y.-I. Hoshino M. (2003). The *in vivo* roles of STEF/Tiam1, Rac1 and JNK in cortical neuronal migration. EMBO J. 22, 4190–4201. 10.1093/emboj/cdg413 12912917 PMC175802

[B94] Keays D. A. Cleak J. Huang G.-J. Edwards A. Braun A. Treiber C. D.. (2010). The role of Tuba1a in adult hippocampal neurogenesis and the formation of the dentate gyrus. Dev. Neurosci. 32, 268–277. 10.1159/000319663 21041996

[B95] Keays D. A. Tian G. Poirier K. Huang G.-J. Siebold C. Cleak J.. (2007). Mutations in α-tubulin cause abnormal neuronal migration in mice and lissencephaly in humans. Cell 128, 45–57. 10.1016/j.cell.2006.12.017 17218254 PMC1885944

[B96] Kerjan G. Gleeson J. G. (2007). Genetic mechanisms underlying abnormal neuronal migration in classical lissencephaly. Trends Genet. 23, 623–630. 10.1016/j.tig.2007.09.003 17997185

[B97] Ketschek A. Spillane M. Dun X.-P. Hardy H. Chilton J. Gallo G. (2016). Drebrin coordinates the actin and microtubule cytoskeleton during the initiation of axon collateral branches. Dev. Neurobiol. 76, 1092–1110. 10.1002/dneu.22377 26731339 PMC4935653

[B98] Kirkcaldie M. T. K. Dwyer S. T. (2017). The third wave: intermediate filaments in the maturing nervous system. Mol. Cell. Neurosci. 84, 68–76. 10.1016/j.mcn.2017.05.010 28554564

[B99] Kitagawa D. Kohlmaier G. Keller D. Strnad P. Balestra F. R. Flückiger I.. (2011). Spindle positioning in human cells relies on proper centriole formation and on the microcephaly proteins CPAP and STIL. J. Cell. Sci. 124, 3884–3893. 10.1242/jcs.089888 22100914

[B100] Koester M. P. Müller O. Pollerberg G. E. (2007). Adenomatous polyposis coli is differentially distributed in growth cones and modulates their steering. J. Neurosci. 27, 12590–12600. 10.1523/JNEUROSCI.2250-07.2007 18003838 PMC6673337

[B101] Koleske A. J. (2013). Molecular mechanisms of dendrite stability. Nat. Rev. Neurosci. 14, 536–550. 10.1038/nrn3486 23839597 PMC3947514

[B102] Kondo M. Takei Y. Hirokawa N. (2012). Motor protein KIF1A is essential for hippocampal synaptogenesis and learning enhancement in an enriched environment. Neuron 73, 743–757. 10.1016/j.neuron.2011.12.020 22365548

[B103] Kouprina N. Pavlicek A. Collins N. K. Nakano M. Noskov V. N. Ohzeki J.-I.. (2005). The microcephaly ASPM gene is expressed in proliferating tissues and encodes for a mitotic spindle protein. Hum. Mol. Genet. 14, 2155–2165. 10.1093/hmg/ddi220 15972725

[B104] Kuijpers M. Hoogenraad C. C. (2011). Centrosomes, microtubules and neuronal development. Mol. Cell. Neurosci. 48, 349–358. 10.1016/j.mcn.2011.05.004 21722732

[B105] Kumar A. Girimaji S. C. Duvvari M. R. Blanton S. H. (2009). Mutations in STIL, encoding a pericentriolar and centrosomal protein, cause primary microcephaly. Am. J. Hum. Genet. 84, 286–290. 10.1016/j.ajhg.2009.01.017 19215732 PMC2668020

[B106] Larti F. Kahrizi K. Musante L. Hu H. Papari E. Fattahi Z.. (2015). A defect in the CLIP1 gene (CLIP-170) can cause autosomal recessive intellectual disability. Eur. J. Hum. Genet. 23, 331–336. 10.1038/ejhg.2014.13 24569606 PMC4326716

[B107] Lee H. Engel U. Rusch J. Scherrer S. Sheard K. Van Vactor D. (2004). The microtubule plus end tracking protein Orbit/MAST/CLASP acts downstream of the tyrosine kinase Abl in mediating axon guidance. Neuron 42, 913–926. 10.1016/j.neuron.2004.05.020 15207236

[B108] Lee J.-R. Srour M. Kim D. Hamdan F. F. Lim S.-H. Brunel-Guitton C.. (2015). *De novo* mutations in the motor domain of KIF1A cause cognitive impairment, spastic paraparesis, axonal neuropathy, and cerebellar atrophy. Hum. Mutat. 36, 69–78. 10.1002/humu.22709 25265257

[B109] Leo L. Yu W. D’Rozario M. Waddell E. A. Marenda D. R. Baird M. A.. (2015). Vertebrate fidgetin restrains axonal growth by severing labile domains of microtubules. Cell Rep. 12, 1723–1730. 10.1016/j.celrep.2015.08.017 26344772 PMC4837332

[B111] Li L. Fothergill T. Hutchins B. I. Dent E. W. Kalil K. (2014). Wnt5a evokes cortical axon outgrowth and repulsive guidance by tau mediated reorganization of dynamic microtubules. Dev. Neurobiol. 74, 797–817. 10.1002/dneu.22102 23818454 PMC4087151

[B110] Li J. Lee W.-L. Cooper J. A. (2005). NudEL targets dynein to microtubule ends through LIS1. Nat. Cell Biol. 7, 686–690. 10.1038/ncb1273 15965467 PMC1569433

[B112] Lin S. Liu M. Mozgova O. I. Yu W. Baas P. W. (2012). Mitotic motors coregulate microtubule patterns in axons and dendrites. J. Neurosci. 32, 14033–14049. 10.1523/JNEUROSCI.3070-12.2012 23035110 PMC3482493

[B113] Liu Y. Lee J. W. Ackerman S. L. (2015). Mutations in the microtubule-associated protein 1A (Map1a) gene cause Purkinje cell degeneration. J. Neurosci. 35, 4587–4598. 10.1523/JNEUROSCI.2757-14.2015 25788676 PMC4363387

[B114] Lizarraga S. B. Margossian S. P. Harris M. H. Campagna D. R. Han A.-P. Blevins S.. (2010). Cdk5rap2 regulates centrosome function and chromosome segregation in neuronal progenitors. Development 137, 1907–1917. 10.1242/dev.040410 20460369 PMC2867323

[B116] Lowery L. A. Stout A. Faris A. E. Ding L. Baird M. A. Davidson M. W.. (2013). Growth cone-specific functions of XMAP215 in restricting microtubule dynamics and promoting axonal outgrowth. Neural Dev. 8:22. 10.1186/1749-8104-8-22 24289819 PMC3907036

[B115] Lowery L. A. Van Vactor D. (2009). The trip of the tip: understanding the growth cone machinery. Nat. Rev. Mol. Cell Biol. 10, 332–343. 10.1038/nrm2679 19373241 PMC2714171

[B117] Lu W. Fox P. Lakonishok M. Davidson M. W. Gelfand V. I. (2013). Initial neurite outgrowth in *Drosophila* neurons is driven by kinesin-powered microtubule sliding. Curr. Biol. 23, 1018–1023. 10.1016/j.cub.2013.04.050 23707427 PMC3676710

[B118] Lucaj C. M. Evans M. F. Nwagbara B. U. Ebbert P. T. Baker C. C. Volk J. G.. (2015). Xenopus TACC1 is a microtubule plus-end tracking protein that can regulate microtubule dynamics during embryonic development. Cytoskeleton 72, 225–234. 10.1002/cm.21224 26012630 PMC4520305

[B119] Maday S. Twelvetrees A. E. Moughamian A. J. Holzbaur E. L. F. (2014). Axonal transport: cargo-specific mechanisms of motility and regulation. Neuron 84, 292–309. 10.1016/j.neuron.2014.10.019 25374356 PMC4269290

[B120] Mandel S. Rechavi G. Gozes I. (2007). Activity-dependent neuroprotective protein (ADNP) differentially interacts with chromatin to regulate genes essential for embryogenesis. Dev. Biol. 303, 814–824. 10.1016/j.ydbio.2006.11.039 17222401

[B121] Mandelkow E.-M. Thies E. Trinczek B. Biernat J. Mandelkow E. (2004). MARK/PAR1 kinase is a regulator of microtubule-dependent transport in axons. J. Cell Biol. 167, 99–110. 10.1083/jcb.200401085 15466480 PMC2172520

[B122] Mao C.-X. Xiong Y. Xiong Z. Wang Q. Zhang Y. Q. Jin S. (2014). Microtubule-severing protein Katanin regulates neuromuscular junction development and dendritic elaboration in *Drosophila*. Development 141, 1064–1074. 10.1242/dev.097774 24550114

[B123] Marx A. Godinez W. J. Tsimashchuk V. Bankhead P. Rohr K. Engel U. (2013). Xenopus cytoplasmic linker-associated protein 1 (XCLASP1) promotes axon elongation and advance of pioneer microtubules. Mol. Biol. Cell 24, 1544–1558. 10.1091/mbc.E12-08-0573 23515224 PMC3655815

[B124] Matamoros A. J. Baas P. W. (2016). Microtubules in health and degenerative disease of the nervous system. Brain Res. Bull. 126, 217–225. 10.1016/j.brainresbull.2016.06.016 27365230 PMC5079814

[B125] Mattila P. K. Lappalainen P. (2008). Filopodia: molecular architecture and cellular functions. Nat. Rev. Mol. Cell Biol. 9, 446–454. 10.1038/nrm2406 18464790

[B126] Maussion G. Carayol J. Lepagnol-Bestel A.-M. Tores F. Loe-Mie Y. Milbreta U.. (2008). Convergent evidence identifying MAP/microtubule affinity-regulating kinase 1 (MARK1) as a susceptibility gene for autism. Hum. Mol. Genet. 17, 2541–2551. 10.1093/hmg/ddn154 18492799

[B127] McNally K. P. Bazirgan O. A. McNally F. J. (2000). Two domains of p80 katanin regulate microtubule severing and spindle pole targeting by p60 katanin. J. Cell. Sci. 113, 1623–1633. Available online at: https://www.ncbi.nlm.nih.gov/pubmed/?term=Two+domains+of+p80+katanin+regulate+microtubule+severing+and+spindle+pole+targeting+by+p60+katanin 10751153 10.1242/jcs.113.9.1623

[B128] McNeely K. C. Cupp T. D. Little J. N. Janisch K. M. Shrestha A. Dwyer N. D. (2017). Mutation of Kinesin-6 Kif20b causes defects in cortical neuron polarization and morphogenesis. Neural Dev. 12:5. 10.1186/s13064-017-0082-5 28359322 PMC5374676

[B129] McVicker D. P. Awe A. M. Richters K. E. Wilson R. L. Cowdrey D. A. Hu X.. (2016). Transport of a kinesin-cargo pair along microtubules into dendritic spines undergoing synaptic plasticity. Nat. Commun. 7:12741. 10.1038/ncomms12741 27658622 PMC5411814

[B130] Menon S. Gupton S. L. (2016). Building blocks of functioning brain: cytoskeletal dynamics in neuronal development. Int. Rev. Cell Mol. Biol. 322, 183–245. 10.1016/bs.ircmb.2015.10.002 26940519 PMC4809367

[B131] Merriam E. B. Lumbard D. C. Viesselmann C. Ballweg J. Stevenson M. Pietila L.. (2011). Dynamic microtubules promote synaptic NMDA receptor-dependent spine enlargement. PLoS One 6:e27688. 10.1371/journal.pone.0027688 22096612 PMC3214068

[B132] Merriam E. B. Millette M. Lumbard D. C. Saengsawang W. Fothergill T. Hu X.. (2013). Synaptic regulation of microtubule dynamics in dendritic spines by calcium, F-actin, and drebrin. J. Neurosci. 33, 16471–16482. 10.1523/JNEUROSCI.0661-13.2013 24133252 PMC3797370

[B133] Mitchison T. Kirschner M. (1984). Dynamic instability of microtubule growth. Nature 312, 237–242. 10.1038/312237a0 6504138

[B134] Moughamian A. J. Osborn G. E. Lazarus J. E. Maday S. Holzbaur E. L. F. (2013). Ordered recruitment of dynactin to the microtubule plus-end is required for efficient initiation of retrograde axonal transport. J. Neurosci. 33, 13190–13203. 10.1523/JNEUROSCI.0935-13.2013 23926272 PMC3735891

[B500] Moores C. A. Perdeeriset M. Francis F. Chelly J. Houdusse A. Milligan R. A. (2004). Mechanism of microtubule stabilization by doublecortin. Mol. Cell 14, 833–839. 10.1016/j.molcel.2004.06.009 15200960

[B135] Mukherjee S. Diaz Valencia J. D. Stewman S. Metz J. Monnier S. Rath U.. (2012). Human Fidgetin is a microtubule severing the enzyme and minus-end depolymerase that regulates mitosis. Cell Cycle 11, 2359–2366. 10.4161/cc.20849 22672901 PMC3383595

[B136] Nakata T. Hirokawa N. (2003). Microtubules provide directional cues for polarized axonal transport through interaction with kinesin motor head. J. Cell Biol. 162, 1045–1055. 10.1083/jcb.200302175 12975348 PMC2172855

[B137] Nakata T. Niwa S. Okada Y. Perez F. Hirokawa N. (2011). Preferential binding of a kinesin-1 motor to GTP-tubulin-rich microtubules underlies polarized vesicle transport. J. Cell Biol. 194, 245–255. 10.1083/jcb.201104034 21768290 PMC3144414

[B138] Neukirchen D. Bradke F. (2011). Cytoplasmic linker proteins regulate neuronal polarization through microtubule and growth cone dynamics. J. Neurosci. 31, 1528–1538. 10.1523/JNEUROSCI.3983-10.2011 21273437 PMC6623617

[B139] Nwagbara B. U. Faris A. E. Bearce E. A. Erdogan B. Ebbert P. T. Evans M. F.. (2014). TACC3 is a microtubule plus end-tracking protein that promotes axon elongation and also regulates microtubule plus end dynamics in multiple embryonic cell types. Mol. Biol. Cell 25, 3350–3362. 10.1091/mbc.E14-06-1121 25187649 PMC4214782

[B140] Ohba C. Haginoya K. Osaka H. Kubota K. Ishiyama A. Hiraide T.. (2015). *De novo* KIF1A mutations cause intellectual deficit, cerebellar atrophy, lower limb spasticity and visual disturbance. J. Hum. Genet. 60, 739–742. 10.1038/jhg.2015.108 26354034

[B141] Oksenberg N. Stevison L. Wall J. D. Ahituv N. (2013). Function and regulation of AUTS2, a gene implicated in autism and human evolution. PLoS Genet. 9:e1003221. 10.1371/journal.pgen.1003221 23349641 PMC3547868

[B143] Ori-McKenney K. M. Jan L. Y. Jan Y. N. (2012). Golgi outposts shape dendrite morphology by functioning as sites of acentrosomal microtubule nucleation in neurons. Neuron 76, 921–930. 10.1016/j.neuron.2012.10.008 23217741 PMC3523279

[B142] Ori-McKenney K. M. Vallee R. B. (2011). Neuronal migration defects in the Loa dynein mutant mouse. Neural Dev. 6:26. 10.1186/1749-8104-6-26 21612657 PMC3127822

[B144] Oz S. Ivashko-Pachima Y. Gozes I. (2012). The ADNP derived peptide, NAP modulates the tubulin pool: implication for neurotrophic and neuroprotective activities. PLoS One 7:e51458. 10.1371/journal.pone.0051458 23272107 PMC3522725

[B145] Oz S. Kapitansky O. Ivashco-Pachima Y. Malishkevich A. Giladi E. Skalka N.. (2014). The NAP motif of activity-dependent neuroprotective protein (ADNP) regulates dendritic spines through microtubule end binding proteins. Mol. Psychiatry 19, 1115–1124. 10.1038/mp.2014.97 25178163

[B146] Pacheco A. Gallo G. (2016). Actin filament-microtubule interactions in axon initiation and branching. Brain Res. Bull. 126, 300–310. 10.1016/j.brainresbull.2016.07.013 27491623 PMC5518172

[B147] Paylor R. Hirotsune S. Gambello M. J. Yuva-Paylor L. Crawley J. N. Wynshaw-Boris A. (1999). Impaired learning and motor behavior in heterozygous Pafah1b1 (Lis1) mutant mice. Learn. Mem. 6, 521–537. 10.1101/lm.6.5.521 10541472 PMC311310

[B148] Pilz D. T. Matsumoto N. Minnerath S. Mills P. Gleeson J. G. Allen K. M.. (1998). LIS1 and XLIS (DCX) mutations cause most classical lissencephaly, but different patterns of malformation. Hum. Mol. Genet. 7, 2029–2037. 10.1093/hmg/7.13.2029 9817918

[B149] Pinto D. Delaby E. Merico D. Barbosa M. Merikangas A. Klei L.. (2014). Convergence of genes and cellular pathways dysregulated in autism spectrum disorders. Am. J. Hum. Genet. 94, 677–694. 10.1016/j.ajhg.2014.03.018 24768552 PMC4067558

[B150] Poirier K. Keays D. A. Francis F. Saillour Y. Bahi N. Manouvrier S.. (2007). Large spectrum of lissencephaly and pachygyria phenotypes resulting from *de novo* missense mutations in tubulin α 1A (TUBA1A). Hum. Mutat. 28, 1055–1064. 10.1002/humu.20572 17584854

[B151] Poirier K. Lebrun N. Broix L. Tian G. Saillour Y. Boscheron C.. (2013a). Mutations in TUBG1, DYNC1H1, KIF5C and KIF2A cause malformations of cortical development and microcephaly. Nat. Genet. 45, 639–647. 10.1038/ng.2613 23603762 PMC3826256

[B153] Poirier K. Saillour Y. Fourniol F. Francis F. Souville I. Valence S.. (2013b). Expanding the spectrum of TUBA1A-related cortical dysgenesis to Polymicrogyria. Eur. J. Hum. Genet. 21, 381–385. 10.1038/ejhg.2012.195 22948023 PMC3598328

[B152] Poirier K. Saillour Y. Bahi-Buisson N. Jaglin X. H. Fallet-Bianco C. Nabbout R.. (2010). Mutations in the neuronal ß-tubulin subunit TUBB3 result in malformation of cortical development and neuronal migration defects. Hum. Mol. Genet. 19, 4462–4473. 10.1093/hmg/ddq377 20829227 PMC3298850

[B155] Poultney C. S. Goldberg A. P. Drapeau E. Kou Y. Harony-Nicolas H. Kajiwara Y.. (2013). Identification of small exonic CNV from whole-exome sequence data and application to autism spectrum disorder. Am. J. Hum. Genet. 93, 607–619. 10.1016/j.ajhg.2013.09.001 24094742 PMC3791269

[B156] Rao A. N. Patil A. Black M. M. Craig E. M. Myers K. A. Yeung H. T.. (2017). Cytoplasmic dynein transports axonal microtubules in a polarity-sorting manner. Cell Rep. 19, 2210–2219. 10.1016/j.celrep.2017.05.064 28614709 PMC5523108

[B157] Rehberg M. Kleylein-Sohn J. Faix J. Ho T.-H. Schulz I. Gräf R. (2005). Dictyostelium LIS1 is a centrosomal protein required for microtubule/cell cortex interactions, nucleus/centrosome linkage, and actin dynamics. Mol. Biol. Cell 16, 2759–2771. 10.1091/mbc.E05-01-0069 15800059 PMC1142422

[B158] Reiner O. Carrozzo R. Shen Y. Wehnert M. Faustinella F. Dobyns W. B.. (1993). Isolation of a Miller-Dieker lissencephaly gene containing G protein β-subunit-like repeats. Nature 364, 717–721. 10.1038/364717a0 8355785

[B159] Rutherford E. L. Carandang L. Ebbert P. T. Mills A. N. Bowers J. T. Lowery L. A. (2016). Xenopus TACC2 is a microtubule plus end-tracking protein that can promote microtubule polymerization during embryonic development. Mol. Biol. Cell 27, 3013–3020. 10.1091/mbc.E16-03-0198 27559128 PMC5063610

[B160] Sainath R. Gallo G. (2015). Cytoskeletal and signaling mechanisms of neurite formation. Cell Tissue Res. 359, 267–278. 10.1007/s00441-014-1955-0 25080065 PMC4286448

[B161] Sapir T. Cahana A. Seger R. Nekhai S. Reiner O. (1999). LIS1 is a microtubule-associated phosphoprotein. Eur. J. Biochem. 265, 181–188. 10.1046/j.1432-1327.1999.00711.x 10491172

[B162] Sapir T. Elbaum M. Reiner O. (1997). Reduction of microtubule catastrophe events by LIS1, platelet-activating factor acetylhydrolase subunit. EMBO J. 16, 6977–6984. 10.1093/emboj/16.23.6977 9384577 PMC1170301

[B163] Sapir T. Frotscher M. Levy T. Mandelkow E.-M. Reiner O. (2012). Tau’s role in the developing brain: implications for intellectual disability. Hum. Mol. Genet. 21, 1681–1692. 10.1093/hmg/ddr603 22194194

[B164] Sapir T. Sapoznik S. Levy T. Finkelshtein D. Shmueli A. Timm T.. (2008). Accurate balance of the polarity kinase MARK2/Par-1 is required for proper cortical neuronal migration. J. Neurosci. 28, 5710–5720. 10.1523/JNEUROSCI.0911-08.2008 18509032 PMC6670809

[B165] Schaar B. T. Kinoshita K. McConnell S. K. (2004). Doublecortin microtubule affinity is regulated by a balance of kinase and phosphatase activity at the leading edge of migrating neurons. Neuron 41, 203–213. 10.1016/s0896-6273(03)00843-2 14741102

[B166] Schirer Y. Malishkevich A. Ophir Y. Lewis J. Giladi E. Gozes I. (2014). Novel marker for the onset of frontotemporal dementia: early increase in activity-dependent neuroprotective protein (ADNP) in the face of Tau mutation. PLoS One 9:e87383. 10.1371/journal.pone.0087383 24489906 PMC3906161

[B167] Sharp D. J. Yu W. Baas P. W. (1995). Transport of dendritic microtubules establishes their nonuniform polarity orientation. J. Cell Biol. 130, 93–103. 10.1083/jcb.130.1.93 7790380 PMC2120517

[B168] Sharp D. J. Yu W. Ferhat L. Kuriyama R. Rueger D. C. Baas P. W. (1997). Identification of a microtubule-associated motor protein essential for dendritic differentiation. J. Cell Biol. 138, 833–843. 10.1083/jcb.138.4.833 9265650 PMC2138050

[B169] Shi S.-H. Cheng T. Jan L. Y. Jan Y. N. (2004). APC and GSK-3β are involved in mPar3 targeting to the nascent axon and establishment of neuronal polarity. Curr. Biol. 14, 2025–2032. 10.1016/j.cub.2004.11.009 15556865

[B170] Shu T. Ayala R. Nguyen M.-D. Xie Z. Gleeson J. G. Tsai L.-H. (2004). Ndel1 operates in a common pathway with LIS1 and cytoplasmic dynein to regulate cortical neuronal positioning. Neuron 44, 263–277. 10.1016/j.neuron.2004.09.030 15473966

[B171] Smith D. S. Niethammer M. Ayala R. Zhou Y. Gambello M. J. Wynshaw-Boris A.. (2000). Regulation of cytoplasmic dynein behaviour and microtubule organization by mammalian Lis1. Nat. Cell Biol. 2, 767–775. 10.1038/35041000 11056530

[B172] Srivastava A. K. Schwartz C. E. (2014). Intellectual disability and autism spectrum disorders: causal genes and molecular mechanisms. Neurosci. Biobehav. Rev. 46, 161–174. 10.1016/j.neubiorev.2014.02.015 24709068 PMC4185273

[B173] Steindler C. Li Z. Algarté M. Alcover A. Libri V. Ragimbeau J.. (2004). Jamip1 (marlin-1) defines a family of proteins interacting with janus kinases and microtubules. J. Biol. Chem. 279, 43168–43177. 10.1074/jbc.M401915200 15277531

[B174] Stiess M. Bradke F. (2011). Neuronal polarization: the cytoskeleton leads the way. Dev. Neurobiol. 71, 430–444. 10.1002/dneu.20849 21557499

[B175] Stiess M. Maghelli N. Kapitein L. C. Gomis-Rüth S. Wilsch-Bräuninger M. Hoogenraad C. C.. (2010). Axon extension occurs independently of centrosomal microtubule nucleation. Science 327, 704–707. 10.1126/science.1182179 20056854

[B176] Stouffer M. A. Golden J. A. Francis F. (2016). Neuronal migration disorders: focus on the cytoskeleton and epilepsy. Neurobiol. Dis. 92, 18–45. 10.1016/j.nbd.2015.08.003 26299390 PMC6508100

[B177] Stutterd C. A. Leventer R. J. (2014). Polymicrogyria: a common and heterogeneous malformation of cortical development. Am. J. Med. Genet. C Semin Med. Genet. 166C, 227–239. 10.1002/ajmg.c.31399 24888723

[B178] Swiech L. Blazejczyk M. Urbanska M. Pietruszka P. Dortland B. R. Malik A. R.. (2011). CLIP-170 and IQGAP1 cooperatively regulate dendrite morphology. J. Neurosci. 31, 4555–4568. 10.1523/JNEUROSCI.6582-10.2011 21430156 PMC6622897

[B179] Takei Y. Kikkawa Y. S. Atapour N. Hensch T. K. Hirokawa N. (2015). Defects in synaptic plasticity, reduced NMDA-receptor transport and instability of postsynaptic density proteins in mice lacking microtubule-associated protein 1A. J. Neurosci. 35, 15539–15554. 10.1523/JNEUROSCI.2671-15.2015 26609151 PMC6705472

[B180] Takei Y. Teng J. Harada A. Hirokawa N. (2000). Defects in axonal elongation and neuronal migration in mice with disrupted tau and map1b genes. J. Cell Biol. 150, 989–1000. 10.1083/jcb.150.5.989 10973990 PMC2175245

[B181] Tanaka E. M. Kirschner M. W. (1991). Microtubule behavior in the growth cones of living neurons during axon elongation. J. Cell Biol. 115, 345–363. 10.1083/jcb.115.2.345 1918145 PMC2289161

[B182] Tanaka T. Koizumi H. Gleeson J. G. (2006). The doublecortin and doublecortin-like kinase 1 genes cooperate in murine hippocampal development. Cereb. Cortex 16, i69–i73. 10.1093/cercor/bhk005 16766710

[B183] Tas R. P. Chazeau A. Cloin B. M. C. Lambers M. L. A. Hoogenraad C. C. Kapitein L. C. (2017). Differentiation between oppositely oriented microtubules controls polarized neuronal transport. Neuron 96, 1264.e5–1271.e5. 10.1016/j.neuron.2017.11.018 29198755 PMC5746200

[B184] Teng J. Takei Y. Harada A. Nakata T. Chen J. Hirokawa N. (2001). Synergistic effects of MAP2 and MAP1B knockout in neuronal migration, dendritic outgrowth, and microtubule organization. J. Cell Biol. 155, 65–76. 10.1083/jcb.200106025 11581286 PMC2150794

[B185] Tint I. Jean D. Baas P. W. Black M. M. (2009). Doublecortin associates with microtubules preferentially in regions of the axon displaying actin-rich protrusive structures. J. Neurosci. 29, 10995–11010. 10.1523/JNEUROSCI.3399-09.2009 19726658 PMC2757270

[B186] Tischfield M. A. Baris H. N. Wu C. Rudolph G. Van Maldergem L. He W.. (2010). Human TUBB3 mutations perturb microtubule dynamics, kinesin interactions, and axon guidance. Cell 140, 74–87. 10.1016/j.cell.2009.12.011 20074521 PMC3164117

[B187] Trimborn M. Bell S. M. Felix C. Rashid Y. Jafri H. Griffiths P. D.. (2004). Mutations in microcephalin cause aberrant regulation of chromosome condensation. Am. J. Hum. Genet. 75, 261–266. 10.1086/422855 15199523 PMC1216060

[B188] Tsai J.-W. Bremner K. H. Vallee R. B. (2007). Dual subcellular roles for LIS1 and dynein in radial neuronal migration in live brain tissue. Nat. Neurosci. 10, 970–979. 10.1038/nn1934 17618279

[B189] Tymanskyj S. R. Scales T. M. E. Gordon-Weeks P. R. (2012). MAP1B enhances microtubule assembly rates and axon extension rates in developing neurons. Mol. Cell. Neurosci. 49, 110–119. 10.1016/j.mcn.2011.10.003 22033417

[B190] Vagnoni A. Hoffmann P. C. Bullock S. L. (2016). Reducing Lissencephaly-1 levels augments mitochondrial transport and has a protective effect in adult *Drosophila* neurons. J. Cell. Sci. 129, 178–190. 10.1242/jcs.179184 26598558 PMC4732301

[B191] Vaillant A. R. Müller R. Langkopf A. Brown D. L. (1998). Characterization of the microtubule-binding domain of microtubule-associated protein 1A and its effects on microtubule dynamics. J. Biol. Chem. 273, 13973–13981. 10.1074/jbc.273.22.13973 9593747

[B192] van de Willige D. Hoogenraad C. C. Akhmanova A. (2016). Microtubule plus-end tracking proteins in neuronal development. Cell. Mol. Life Sci. 73, 2053–2077. 10.1007/s00018-016-2168-3 26969328 PMC4834103

[B193] Van Dijck A. Vulto-van Silfhout A. T. Cappuyns E. van der Werf I. M. Mancini G. M. Tzschach A.. (2018). Clinical presentation of a complex neurodevelopmental disorder caused by mutations in ADNP. Biol. Psychiatry [Epub ahead of print]. 10.1016/j.biopsych.2018.02.1173 29724491 PMC6139063

[B194] Vidal R. L. Ramírez O. A. Sandoval L. Koenig-Robert R. Härtel S. Couve A. (2007). Marlin-1 and conventional kinesin link GABAB receptors to the cytoskeleton and regulate receptor transport. Mol. Cell. Neurosci. 35, 501–512. 10.1016/j.mcn.2007.04.008 17532644

[B195] Vulih-Shultzman I. Pinhasov A. Mandel S. Grigoriadis N. Touloumi O. Pittel Z.. (2007). Activity-dependent neuroprotective protein snippet NAP reduces tau hyperphosphorylation and enhances learning in a novel transgenic mouse model. J. Pharmacol. Exp. Ther. 323, 438–449. 10.1124/jpet.107.129551 17720885

[B196] Vulprecht J. David A. Tibelius A. Castiel A. Konotop G. Liu F.. (2012). STIL is required for centriole duplication in human cells. J. Cell. Sci. 125, 1353–1362. 10.1242/jcs.104109 22349705

[B197] Wang Y. Mandelkow E. (2016). Tau in physiology and pathology. Nat. Rev. Neurosci. 17, 22–35. 10.1038/nrn.2015.1 26631930

[B198] Whitman M. C. Andrews C. Chan W.-M. Tischfield M. A. Stasheff S. F. Brancati F.. (2016). Two unique TUBB3 mutations cause both CFEOM3 and malformations of cortical development. Am. J. Med. Genet. A 170A, 297–305. 10.1002/ajmg.a.37362 26639658 PMC4770801

[B199] Willemsen M. H. Ba W. Wissink-Lindhout W. M. de Brouwer A. P. M. Haas S. A. Bienek M.. (2014). Involvement of the kinesin family members KIF4A and KIF5C in intellectual disability and synaptic function. J. Med. Genet. 51, 487–494. 10.1136/jmedgenet-2013-102182 24812067

[B200] Winding M. Kelliher M. T. Lu W. Wildonger J. Gelfand V. I. (2016). Role of kinesin-1-based microtubule sliding in *Drosophila* nervous system development. Proc. Natl. Acad. Sci. U S A 113, E4985–E4994. 10.1126/science.353.6302.883-a 27512046 PMC5003282

[B201] Witte H. Neukirchen D. Bradke F. (2008). Microtubule stabilization specifies initial neuronal polarization. J. Cell Biol. 180, 619–632. 10.1083/jcb.200707042 18268107 PMC2234250

[B202] Wood J. D. Landers J. A. Bingley M. McDermott C. J. Thomas-McArthur V. Gleadall L. J.. (2006). The microtubule-severing protein Spastin is essential for axon outgrowth in the zebrafish embryo. Hum. Mol. Genet. 15, 2763–2771. 10.1093/hmg/ddl212 16893913

[B203] Yau K. W. Schätzle P. Tortosa E. Pagès S. Holtmaat A. Kapitein L. C.. (2016). Dendrites *in vitro* and *in vivo* contain microtubules of opposite polarity and axon formation correlates with uniform plus-end-out microtubule orientation. J. Neurosci. 36, 1071–1085. 10.1523/JNEUROSCI.2430-15.2016 26818498 PMC4728718

[B204] Yau K. W. van Beuningen S. F. B. Cunha-Ferreira I. Cloin B. M. C. van Battum E. Y. Will L.. (2014). Microtubule minus-end binding protein CAMSAP2 controls axon specification and dendrite development. Neuron 82, 1058–1073. 10.1016/j.neuron.2014.04.019 24908486

[B205] Youn Y. H. Pramparo T. Hirotsune S. Wynshaw-Boris A. (2009). Distinct dose-dependent cortical neuronal migration and neurite extension defects in Lis1 and Ndel1 mutant mice. J. Neurosci. 29, 15520–15530. 10.1523/JNEUROSCI.4630-09.2009 20007476 PMC2824645

[B206] Yu W. Ahmad F. J. Baas P. W. (1994). Microtubule fragmentation and partitioning in the axon during collateral branch formation. J. Neurosci. 14, 5872–5884. 10.1523/JNEUROSCI.14-10-05872.1994 7931550 PMC6576981

[B207] Yu W. Cook C. Sauter C. Kuriyama R. Kaplan P. L. Baas P. W. (2000). Depletion of a microtubule-associated motor protein induces the loss of dendritic identity. J. Neurosci. 20, 5782–5791. 10.1523/JNEUROSCI.20-15-05782.2000 10908619 PMC6772545

[B208] Yu W. Qiang L. Solowska J. M. Karabay A. Korulu S. Baas P. W. (2008). The microtubule-severing proteins spastin and katanin participate differently in the formation of axonal branches. Mol. Biol. Cell 19, 1485–1498. 10.1091/mbc.E07-09-0878 18234839 PMC2291400

[B209] Yuan A. Kumar A. Peterhoff C. Duff K. Nixon R. A. (2008). Axonal transport rates *in vivo* are unaffected by tau deletion or overexpression in mice. Mol. Hum. Reprod. 28, 1682–1687. 10.1523/JNEUROSCI.5242-07.2008 18272688 PMC2814454

[B210] Zhao B. Meka D. P. Scharrenberg R. König T. Schwanke B. Kobler O.. (2017). Microtubules modulate F-actin dynamics during neuronal polarization. Sci. Rep. 7:9583. 10.1038/s41598-017-09832-8 28851982 PMC5575062

[B211] Zhao J. Wang Y. Xu H. Fu Y. Qian T. Bo D.. (2016). Dync1h1 mutation causes proprioceptive sensory neuron loss and impaired retrograde axonal transport of dorsal root ganglion neurons. CNS Neurosci. Ther. 22, 593–601. 10.1111/cns.12552 27080913 PMC6492895

[B212] Zheng Y. Wildonger J. Ye B. Zhang Y. Kita A. Younger S. H.. (2008). Dynein is required for polarized dendritic transport and uniform microtubule orientation in axons. Nat. Cell Biol. 10, 1172–1180. 10.1038/ncb1777 18758451 PMC2588425

[B213] Zollo M. Ahmed M. Ferrucci V. Salpietro V. Asadzadeh F. Carotenuto M.. (2017). PRUNE is crucial for normal brain development and mutated in microcephaly with neurodevelopmental impairment. Brain 140, 940–952. 10.1093/brain/awx014 28334956 PMC5382943

